# Histamine H_1_ receptor deletion in cholinergic neurons induces sensorimotor gating ability deficit and social impairments in mice

**DOI:** 10.1038/s41467-021-21476-x

**Published:** 2021-02-18

**Authors:** Li Cheng, Cenglin Xu, Lu Wang, Dadao An, Lei Jiang, Yanrong Zheng, Yixin Xu, Yi Wang, Yujing Wang, Kuo Zhang, Xiaodong Wang, Xiangnan Zhang, Aimin Bao, Yudong Zhou, Jingyu Yang, Shumin Duan, Dick F. Swaab, Weiwei Hu, Zhong Chen

**Affiliations:** 1 Institute of Pharmacology & Toxicology, NHC and CAMS Key Laboratory of Medical Neurobiology, College of Pharmaceutical Sciences, School of Basic Medical Sciences, Zhejiang University, Hangzhou, Zhejiang P.R. China; 2 Key Laboratory of Neuropharmacology and Translational Medicine of Zhejiang Province, College of Pharmaceutical Sciences, Zhejiang Chinese Medical University, Hangzhou, Zhejiang P.R. China; 3 Department of Pharmacology, Shenyang Pharmaceutical University, Shenyang, P.R. China; 4 Netherlands Institute for Neuroscience, an Institute of the Royal Netherlands Academy of Arts and Sciences, Amsterdam, BA the Netherlands

**Keywords:** Target identification, Schizophrenia, Schizophrenia

## Abstract

Negative symptoms in schizophrenia strongly contribute to poor functional outcomes, however its pathogenesis is still unclear. Here, we found that histamine H_1_ receptor (H_1_R) expression in basal forebrain (BF) cholinergic neurons was decreased in patients with schizophrenia having negative symptoms. Deletion of H_1_R gene in cholinergic neurons in mice resulted in functional deficiency of cholinergic projections from the BF to the prefrontal cortex and in the formation of sensorimotor gating deficit, social impairment and anhedonia-like behavior. These behavioral deficits can be rescued by re-expressing H_1_R or by chemogenetic activation of cholinergic neurons in the BF. Direct chemogenetic inhibition of BF cholinergic neurons produced such behavioral deficits and also increased the susceptibility to hyperlocomotion. Our results suggest that the H_1_R deficiency in BF cholinergic neurons is critical for sensorimotor gating deficit, social impairments and anhedonia-like behavior. This finding may help to understand the genetic and biochemical bases of negative symptoms in schizophrenia.

## Abstract (web summary)

Social impairment and anhedonia are common negative symptoms in patients with schizophrenia. Here, the authors show that the histamine H_1_ receptor in cholinergic neurons in the basal forebrain has a critical role in sensorimotor gating, social behaviour, and anhedonia-like behaviour in mice.

## Introduction

Schizophrenia typically manifests positive symptoms including delusions, hallucinations, and disordered thoughts, which tend to decrease over time; negative symptoms including blunted affect, speech poverty, social withdrawal, and anhedonia, which tend to remain stable or increase over time; as well as cognitive impairments^1,2^. Notably, resolution of positive symptoms does not necessarily translate to functional recovery, while negative symptoms strongly contribute to poor functional outcomes^3,4^. Currently, routine clinical used antipsychotics that block dopamine D2 receptors and serotonin receptors are not highly-efficient or exclusive for the treatment of negative symptoms. Treatments that target negative symptoms remain an unmet need.

Biological and cellular evidence supported that a deficit of glutamate transmission in the prefrontal cortex (PFC) via N-methyl-D-aspartate receptors (NMDARs) and subsequently reduced mesocortical dopaminergic activity via D1 receptors are the likely basis for negative symptoms in schizophrenia^4,5^. Glutamatergic antipsychotics, such as d-serine, sarcosine, N-acetyl-cysteine, and D-cycloserine that enhance NMDA receptor function remain under clinical development^6^. In clinical trials, although the effects of glutamatergic antipsychotics on negative symptoms are statistically significant, none reaches the clinically significant threshold for improvement^7,8^. D1 receptor agonist DAR-0100A shows no significant effects on negative symptoms^9^, while modafinil, that more broadly increases dopaminergic activity, provides a significant but small benefit when used adjunctive to treat acute psychotic illness^10^. Although the glutamatergic approaches and other anti-inflammatory or nicotinic alpha-7 related agents continue to hold promise, the identification of the exact neuronal function that is critically affected in schizophrenia and what patterns of disconnection lead to the expression of negative symptoms may facilitate the development of novel therapeutic targets.

The histaminergic neuron is known to hold a key position in the regulation of sleep and wakefulness, learning and memory, feeding, and energy homeostasis, primarily through the histamine H_1_ receptor (H_1_R)^11,12^. Recent studies suggest that histamine or its receptor is linked to psychological disorders such as schizophrenia. The level of tele-methylhistamine, the major histamine metabolite, is elevated in the cerebrospinal fluid (CSF) of patients with schizophrenia who were either taking or withdrawn from the neuroleptic therapy for up to 6 weeks, reflecting increased histamine release and turnover in these patients^13^. The tele-methylhistamine level correlated with the ratio of negative/positive symptom scores in the patients with predominantly negative symptoms^13^. Low H_1_R binding was observed in the frontal and prefrontal cortices and cingulate cortex of people with schizophrenia treated with haloperidol^14^. However, many atypical antipsychotics such as clozapine and olanzapine have high blocking ability for H_1_R^15,16^. It implies that H_1_R may play an intricate role in the schizophrenia.

In present study, we found that patients with schizophrenia having negative symptoms exhibited prominently reduced H_1_R expression in cholinergic neurons. We used the Cre-LoxP system to generate mice with a targeted deletion of H_1_R in glutamatergic neurons (*CaMKIIα-Cre;Hrh1*^*fl/fl*^), dopaminergic neurons (*DAT*^*−*^*Cre;Hrh1*^*fl/fl*^), or cholinergic neurons (*ChAT*^*−*^*Cre;Hrh1*^*fl/fl*^). Interestingly, we found that *ChAT-Cre;Hrh1*^*fl/fl*^ mice, but not *CaMKIIα-Cre;Hrh1*^*fl/fl*^ or *DAT-Cre;Hrh1*^*fl/fl*^ mice, display impaired prepulse inhibition (PPI), abnormal social behaviors, and anhedonia-like behavior. We confirmed that H_1_R in basal forebrain (BF) cholinergic neurons plays a critical role in the pathogenesis of such behavioral deficits and identified the underlying circuit mechanism by selective re-expression of H_1_R in BF cholinergic neurons as well as through chemogenetic approaches to activate or inhibit the BF cholinergic neurons.

## Results

### Decreased histamine H_1_R expression in cholinergic neurons in patients with schizophrenia having negative symptoms

**Fig. 1:**
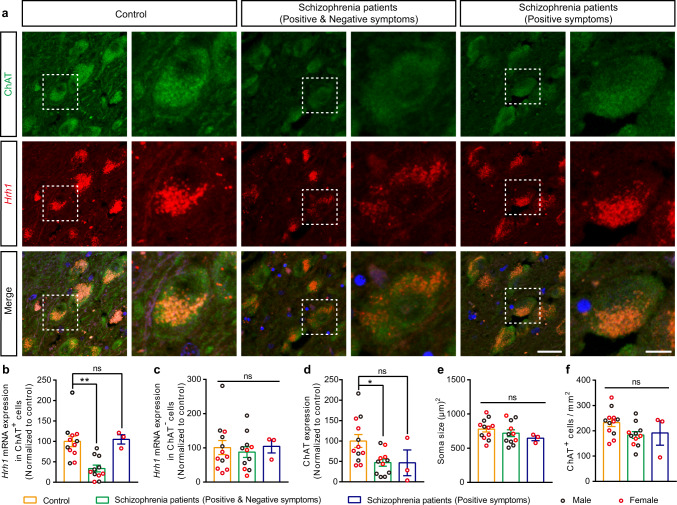
Decreased histamine H_1_R expression in BF cholinergic neurons in patients with schizophrenia having negative symptoms. **a** Representative images of RNAscope in situ hybridization of *Hrh1* mRNA together with immunostaining of choline acetyltransferase (ChAT) in the NBM of BF for patients with schizophrenia and controls. The right panels are enlarged from the dotted box in the left panels. Left: scale bar, 30 μm. Right: scale bar, 10 μm. **b**–**f** *Hrh1* mRNA expression in ChAT^+^ or ChAT^−^cells, ChAT expression, soma size, and density of ChAT^+^ cells in patients with schizophrenia and controls (*n* = 12 for controls, *n* = 11 for patients with schizophrenia having positive and negative symptoms, and *n* = 3 for patients with schizophrenia having positive symptoms). **b** Quantitative analysis of *Hrh1* mRNA expression in ChAT^+^ cells of patients with schizophrenia and controls. **c** Quantitative analysis of *Hrh1* mRNA expression in ChAT^-^ cells of patients with schizophrenia and controls. **d** Quantitative analysis of ChAT expression of ChAT^+^ cells in patients with schizophrenia and controls. **e** Quantitative analysis of soma size of ChAT^+^ cells in patients with schizophrenia and controls. **f** Quantitative analysis of density of ChAT^+^ cells in patients with schizophrenia and controls. All data are presented as mean ± s.e.m. and error bars represent s.e.m. *P ≤ 0.05, **P ≤ 0.01, ns nonsignificant. See also Supplementary Data 2 for further statistical information. Source data are provided as a Source Data file.

Since the post-mortem and neuroimaging studies reveal that cholinergic dysfunction is observed in individuals with schizophrenia^17,18^, we investigated the level of *Hrh1* mRNA in cholinergic neurons by RNAscope in situ hybridization (ISH) in brain samples from adult patients with schizophrenia (Supplementary Data 1). Compared with age-matched controls, 11 patients with schizophrenia having both positive and negative symptoms, including withdrawn, depressed, passive, or inactive characteristics, exhibited robust low *Hrh1* mRNA expression in the choline acetyltransferase (ChAT) positive cholinergic neurons rather than ChAT negative cells, in the nucleus basalis magnocellularis (NBM) area of BF (Fig. 1a–c). Meanwhile, we observed the decreased ChAT expression in the NBM of BF, while the soma size and cell density of cholinergic neurons were unchanged (Fig. 1d–f). In contrast, three patients with schizophrenia having positive symptom only showed no change in H_1_R expression (Fig. 1a–c). The *Hrh1* mRNA and ChAT expression was further analyzed for males and females, respectively. We found that the male patients with schizophrenia having positive and negative symptoms showed decrease in the *Hrh1* mRNA expression (unpaired two-tailed *t* test, *t* = 2.593, *P* = 0.027). Meanwhile, the female patients with schizophrenia having positive and negative symptoms (*P* < 0.001), but not patients with schizophrenia having positive symptoms only (*P* = 0.560), showed decrease in the *Hrh1* mRNA expression (one-way ANOVA with post hoc Tukey’s test, *F*_2, 11_ = 20.070, *P* = 0.302). Furthermore, the ChAT expression was only significantly reduced in male patients with schizophrenia having positive and negative symptoms (unpaired two-tailed *t* test, *t* = 2.885, *P* = 0.016), but not female patients with schizophrenia having negative symptoms (*P* = 0.667) or not (*P* = 0.381) (one-way ANOVA with post hoc Tukey’s test, *F*_2, 11_ = 1.059, *P* = 0.380). Considering the *n* value of human post-mortem brain tissues is small, more samples are needed to confirm above results in the future. Together, above results implicate that H_1_R in cholinergic neurons may be involved in pathogenesis of schizophrenia including negative symptoms.

### Decreased histamine H_1_R expression in cholinergic neurons induces sensorimotor gating ability deficit, social impairments, and anhedonia-like behavior

To examine the relationship of H_1_R in the cholinergic neurons to schizophrenia, we crossed the *ChAT-Cre* line with *Hrh1*^*fl/fl*^ mice to induce H_1_R deletion in cholinergic neurons. The *ChAT-Cre* mice were firstly crossed with *Ai14* mice to label Cre-positive neurons with red fluorescent protein tdTomato^19^ to determine Cre distribution in the *ChAT-Cre* mouse line. We found that the Cre recombinase was expressed in the cholinergic neurons, but not other types of neurons, in BF (98.85 ± 1.15%, *n* = 3) and caudate putamen (CPu) (100 ± 0%, *n* = 5) in this *ChAT-Cre* mouse line (Supplementary Fig. 1a). The deficiency of H_1_R expression in the cholinergic neurons in *ChAT-Cre;Hrh1*^*fl/fl*^ mice was then validated by RNAscope ISH (Supplementary Fig. 2). The vast majority of ChAT positive cholinergic neurons in different areas in the BF as well as the CPu expressed *Hrh1* mRNA (ranged from 97.3 to 100%) in *Hrh1*^*fl/fl*^ control mice, while about half of cholinergic neurons in these areas (ranged from 48.6 to 69.9%) were absent for the *Hrh1* mRNA expression in the *ChAT-Cre;Hrh1*^*fl/fl*^ mice (Supplementary Fig. 2b, d). Moreover, the *Hrh1* mRNA level in cholinergic neurons was very low in *ChAT-Cre;Hrh1*^*fl/fl*^ mice (Supplementary Fig. 2b, e). It indicates significant deletion of H_1_R from cholinergic neurons with the Cre-LoxP system. Furthermore, frequency distribution of *Hrh1* mRNA expression was analyzed for ChAT^+^ cholinergic cells from *Hrh1*^*fl/fl*^ and *ChAT*-*Cre;Hrh1*^*fl/fl*^ mice (Supplementary Fig. 3). We observed a shift toward lower values for *Hrh1* mRNA expression in the *ChAT-Cre;Hrh1*^*fl/fl*^ mice compared to controls. Among cholinergic neurons in the BF, 85.3% neurons had low level of *Hrh1* mRNA (<1 A.U.) in *ChAT-Cre;Hrh1*^*fl/fl*^, while 92.57% neurons had high level of *Hrh1* mRNA (>1 A.U.) in *Hrh1*^*fl/fl*^ mice. The remaining expression of *Hrh1* mRNA in cholinergic neurons in *ChAT-Cre;Hrh1*^*fl/fl*^ mice may be due to some sort of feedback loop in cells with one allele absent.

Compared with littermates, the *ChAT-Cre;Hrh1*^*fl/fl*^ male mice showed similar basic functional capacity for olfactory function, motor coordination, body growth rate, body temperature, and pain sensitivity (Supplementary Fig. 4a–e). Moreover, *ChAT-Cre;Hrh1*^*fl/fl*^ male mice showed normal locomotor activity, as demonstrated by a similar total distance traveled in the open field tests compared to control mice (Supplementary Fig. 5a–c). The hyperlocomotion elicited by noncompetitive NMDAR antagonist MK-801 has been used as a model of positive symptoms in pharmacological experiments^20^. Here, the locomotor hyperactivity induced by MK-801 appeared in the control mice, however it was attenuated in the mutant mice (Supplementary Fig. 5d, e).

**Fig. 2:**
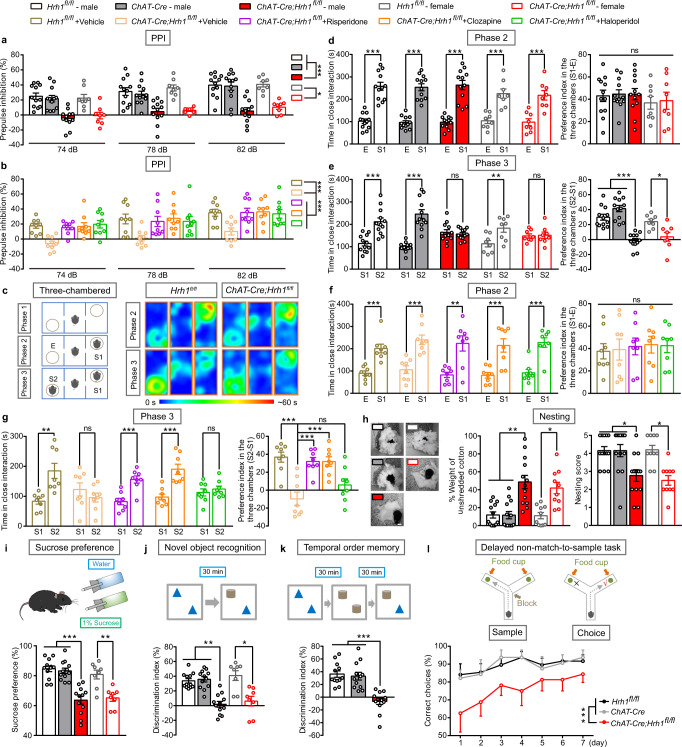
Decreased histamine H_1_R expression in cholinergic neurons induces sensorimotor gating ability deficit, social impairments, anhedonia-like behavior, and cognitive impairments. **a–l** Sensorimotor gating, social behavior, hedonic function, and cognitive behavior in *Hrh1*^*fl/fl*^, *ChAT-Cre,* and *ChAT-Cre;Hrh1*^*fl/fl*^ mice. **a** Percentage of prepulse inhibition of the auditory startle reflex across different prepulse intensities. **b** Percentage of prepulse inhibition of the auditory startle reflex across different prepulse intensities after treatment of antipsychotic agent risperidone, clozapine, or haloperidol. **c** Schematic diagram of three-chamber test and representative heat map images of phase 2 and phase 3. E: empty wire cage; S1: a gender-matched stranger mouse; S2: a new gender-matched stranger mouse. **d** Time in close interaction and preference index during sociability testing (phase 2) when exposed to S1. **e** Time in close interaction and preference index during subsequent social novelty recognition testing (phase 3) when exposed to S2 together with S1. **f** Time in close interaction and preference index of phase 2 in three-chamber test treated with antipsychotic agent risperidone, clozapine, or haloperidol. **g** Time in close interaction and preference index of phase 3 in three-chamber test treated with antipsychotic agent risperidone, clozapine, or haloperidol. **h** The representative images, percentage of the weight of cotton left unshredded, and nesting score in nest-building test. Scale bar, 2 cm. **i** Test for sucrose preference as a percentage of all fluid intake within a 48 h period. **j** Discrimination index in a novel object recognition test, with schematic diagram in the upper panel. **k** Discrimination index in temporal order memory test, with schematic diagram in the upper panel. **l** Percentage of correct choices in choice runs of delayed non-match-to-sample task. All data are presented as mean ± s.e.m. and error bars represent s.e.m. *P ≤ 0.05, **P ≤ 0.01, ***P ≤ 0.001, ns nonsignificant. See also Supplementary Data 2 for further statistical information. Source data are provided as a Source Data file.

Interestingly, selective deletion of H_1_R in cholinergic neurons disrupted PPI of the auditory startle reflex (Fig. 2a), without affecting acoustic startle reactivity (Supplementary Fig. 6a). PPI of the auditory startle reflex is a highly conserved sensorimotor gating measure across mammalian species and PPI deficits have been repeatedly documented in individuals with schizophrenia^21–23^. To ask whether the behavioral deficits of *ChAT-Cre;Hrh1*^*fl/fl*^ mice could be reversed by antipsychotics, we evaluated their behavior following chronic treatment with typical antipsychotic agent haloperidol, and atypical antipsychotic agents risperidone and clozapine. We found that the chronic treatment of anyone of these antipsychotic agents completely rescued the PPI deficit observed in the *ChAT-Cre;Hrh1*^*fl/fl*^ male mice (Fig. 2b). These results indicate that loss of H_1_R in cholinergic neurons impaired sensorimotor gating abilities in PPI, which were relieved by antipsychotic agents.

We further assessed social behavior and hedonic function, dysfunction of which is closely linked to negative symptoms of schizophrenia. Social interactions involving sociability and social novelty recognition were examined in the phase 2 and phase 3 of a three-chamber test (Fig. 2c). Both mutant and control mice showed no preference for the right or left chamber during the habituation period in phase 1 (Supplementary Fig. 6b). When a stranger mouse (S1) was placed in one chamber during phase 2, both mutant and control mice spent more time to closely interact with the strangers, suggesting similar sociability between control and mutant mice (Fig. 2d and Supplementary Fig. 6b). When a second stranger mouse was placed in the chamber on the unoccupied side during phase 3, *ChAT-Cre;Hrh1*^*fl/fl*^ male mice showed reduced preference for the unfamiliar mouse compared with the control mice, indicating reduced social novelty recognition (Fig. 2e and Supplementary Fig. 6b). To study the possibility that the no preference for the novel animal in *ChAT-Cre;Hrh1*^*fl/fl*^ mice may result from their deficit in novelty recognition, we performed a modified novel object recognition test, in which the test was immediately exerted after the free exploration, with a similar time course as the three-chamber test (Supplementary Fig. 6d). In this test, *ChAT-Cre;Hrh1*^*fl/fl*^ mice showed intact novelty recognition ability compared with control mice. It suggests that the no preference for the novel animal in *ChAT-Cre;Hrh1*^*fl/fl*^ mice is related to aberrant social novelty recognition, rather than a general impairment of recognition ability. Furthermore, the administration of antipsychotic agents risperidone and clozapine, but not haloperidol, completely rescued the social novelty recognition deficit observed in the *ChAT-Cre;Hrh1*^*fl/fl*^ mice (Fig. 2f, g). A nest-building test was also employed to assess social activity. Compared to controls, *ChAT-Cre;Hrh1*^*fl/fl*^ male mice were not able to form an identifiable nest, but had a tendency to scatter nesting material, indicating an inability to properly build a nest, a condition associated with poor planning in organized behavior and social withdrawal (Fig. 2h).

To test hedonic function, the sucrose preference test is often conducted to explore the symptom of anhedonia. *ChAT-Cre;Hrh1*^*fl/fl*^ male mice had a decreased preference for sweet solutions but showed no change in total fluid intake, indicating an anhedonia-like state (Fig. 2i and Supplementary Fig. 6c). Although anhedonia and social impairment are also associated with depression, *ChAT-Cre;Hrh1*^*fl/fl*^ mice did not show aberrant features in the tail suspension test and forced swimming test, two classic measures of behavioral despair for rodents that are often used to evaluate the efficacy of antidepressants (Supplementary Fig. 7a, b).

Cognitive deficits, including failures of episodic memory and working memory, have also been suggested as one of the core symptoms of schizophrenia^24,25^. In both regular novel object recognition and temporal order object recognition tasks, control mice spent more time exploring the object presented less recently, while the *ChAT-Cre;Hrh1*^*fl/fl*^ male mice showed no preference for either object (Fig. 2j, k). In contrast to the deficiency in regular novel object recognition test which has 30 min retention interval between free exploration and a subsequent test, *ChAT-Cre;Hrh1*^*fl/fl*^ mice displayed comparable preference for the novel object as controls in a modified novel object recognition, which has no retention between free exploration and a subsequent test. It suggests that *ChAT-Cre;Hrh1*^*fl/fl*^ mice reserve the ability to appreciate and discriminate the novel object^26^. Since the 30 min interval is related to the retention for the short-term memory, the deficits in both regular novel object recognition and temporal order object recognition tasks are linked to impaired episodic memory. We then evaluated working memory of *ChAT-Cre;Hrh1*^*fl/fl*^ male mice by the delayed non-match-to-sample (DNMTS) task in a Y-maze, and found that *ChAT-Cre;Hrh1*^*fl/fl*^ mice showed impaired working memory with a lower percent of correct choices averaged across a given test period (Fig. 2l). However, the *ChAT-Cre;Hrh1*^*fl/fl*^ mice also displayed intact spatial memory in a Morris water maze test (Supplementary Fig. 6e). Light-dark box tests and elevated plus maze (EPM) were also performed to evaluate whether mutant mice display anxiety, which is frequently observed among schizophrenic patients^27^. Compared with control mice, *ChAT-Cre;Hrh1*^*fl/fl*^ male mice spent less time and fewer entries on exploring the open arms in the EPM test (Supplementary Fig. 7d) and less time in the light box in the light-dark box test (Supplementary Fig. 7c). The above data indicate that selective deletion of H_1_R in cholinergic neurons induces impairment in episodic memory and working memory, and anxiety-like behaviors. Moreover, although it has been reported that histamine deficiency caused by loss of function mutation of histidine decarboxylase leads to tic-like stereotypes resembling Tourette syndrome^28,29^, no stereotypic behavior was observed in *ChAT-Cre;Hrh1*^*fl/fl*^ mice through assessment of their digging, grooming, and jumping behaviors (Supplementary Fig. 4f).

Since the aberrant H_1_R expression was observed in both male and female patient, we also examined the behavioral features in female *ChAT-Cre;Hrh1*^*fl/fl*^ mice. Female *ChAT-Cre;Hrh1*^*fl/fl*^ mice displayed sensorimotor gating deficits and social impairments along with the cognitive dysfunction, which are comparable to the male *ChAT-Cre;Hrh1*^*fl/fl*^ mice (Fig. 2a, d, e, h–j). Taken together, the above results further address that ablation of H_1_R in cholinergic neurons results in sensorimotor gating ability deficit, social impairments, and anhedonia-like behavior.

In addition, we also investigated the role of H_1_R in dopaminergic or glutamatergic neurons. We deleted H_1_R from these two neuron types by breeding mice carrying the *Hrh1* allele flanked by loxp sites (*Hrh1*^*fl/fl*^) with *DAT-Cre* or *CaMKIIα-Cre* transgenic mice (Supplementary Fig. 8a, i). RNAscope ISH of H_1_R mRNA together with immunostaining of TH (Tyrosine hydroxylase, a marker for dopaminergic neurons) or glutamate (a marker for glutamatergic neurons) confirmed successful deletion of *Hrh1* from dopaminergic and glutamatergic neurons in *DAT-Cre;Hrh1*^*fl/fl*^ and *CaMKIIα-Cre;Hrh1*^*fl/fl*^ mice, respectively (Supplementary Fig. 8b, j). Both the *DAT-Cre;Hrh1*^*fl/fl*^ and *CaMKIIα-Cre;Hrh1*^*fl/fl*^ mice showed comparable behavior to control mice in PPI, as well as in the open field locomotor test, three-chamber test, nesting test, sucrose preference test, and novel object recognition test (Supplementary Fig. 8c–h, k–p). These results suggest that the deficiency of H_1_R in dopaminergic and glutamatergic neurons does not induce the phenotype as that in mice with deficiency of H_1_R in cholinergic neurons.

### Deletion of H_1_R in cholinergic neurons results in dysfunction of cholinergic neurons in the BF and an excitation/inhibition imbalance in the PFC

**Fig. 3:**
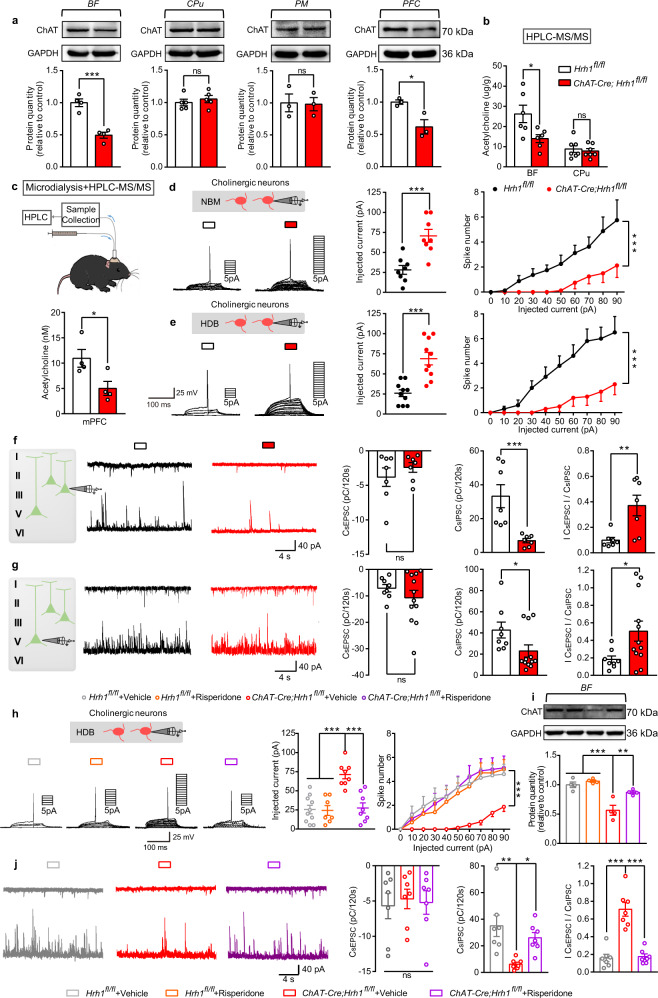
Deletion of H_1_R in cholinergic neurons results in dysfunction of cholinergic neurons in the BF and an excitation/inhibition imbalance in the PFC. **a** Western blot of ChAT in BF, CPu, PM, and PFC. **b** Acetycholine content in BF and CPu examined by HPLC-MS/MS. **c** Extracellular acetycholine level in the medial prefrontal cortex collected by microdialysis and examined by HPLC-MS/MS. **d** Threshold current to elicit action potential with the increase of injected currents in NBM cholinergic neurons recorded by whole-cell patch-clamp. Spike numbers with the increase of injected currents in NBM cholinergic neurons recorded by whole-cell patch-clamp. **e** Threshold current to elicit action potential with the increase of injected currents in HDB cholinergic neurons recorded by whole-cell patch-clamp. Spike numbers with the increase of injected currents in HDB cholinergic neurons recorded by whole-cell patch-clamp. **f** Representative traces showing sEPSC (upper) and sIPSC (lower) recorded at −60 mV (upper) and +10 mV (lower) in the same mPFC layer 2/3 pyramidal cell from *Hrh1*^*fl/fl*^ (black) or *ChAT-Cre;Hrh1*^*fl/fl*^ (red) mice. Quantification of sEPSC and sIPSC charge transfer and sEPSC/sIPSC charge transfer ratios in *Hrh1*^*fl/fl*^ and *ChAT-Cre;Hrh1*^*fl/fl*^ mice. **g** Representative traces showing sEPSC (upper) and sIPSC (lower) recorded at −60 mV (upper) and +10 mV (lower) in the same mPFC layer 5 pyramidal cell from *Hrh1*^*fl/fl*^ (black) or *ChAT-Cre;Hrh1*^*fl/fl*^ (red) mice. Quantification of sEPSC and sIPSC charge transfer and sEPSC/sIPSC charge transfer ratios in *Hrh1*^*fl/fl*^ and *ChAT-Cre;Hrh1*^*fl/fl*^ mice. **h** Threshold current to elicit action potential with the increase of injected currents in HDB cholinergic neurons treated with antipsychotic agent risperidone recorded by whole-cell patch-clamp. Spike numbers with the increase of injected currents in HDB cholinergic neurons treated with antipsychotic agent risperidone recorded by whole-cell patch-clamp. **i** Western blot of choline acetyltransferase (ChAT) treated with antipsychotic agent risperidone in basal forebrain. **j** Representative traces showing sEPSC (upper) and sIPSC (lower) recorded at −60 mV (upper) and +10 mV (lower) in the same mPFC layer 2/3 pyramidal cell from *Hrh1*^*fl/fl*^ + Vehicle (gray), *ChAT-Cre;Hrh1*^*fl/fl*^ + Vehicle (red), or *ChAT-Cre;Hrh1*^*fl/fl*^ treated with antipsychotic agent risperidone (purple). Quantification of sEPSC and sIPSC charge transfer and sEPSC/sIPSC charge transfer ratios in *Hrh1*^*fl/fl*^ + Vehicle, *ChAT-Cre;Hrh1*^*fl/fl*^ + Vehicle, or *ChAT-Cre;Hrh1*^*fl/fl*^ treated with antipsychotic agent risperidone mice. All data are presented as mean ± s.e.m. and error bars represent s.e.m. *P ≤ 0.05, **P ≤ 0.01, ***P ≤ 0.001, ns nonsignificant. See also Supplementary Data 2 for further statistical information. Source data are provided as a Source Data file.

Cholinergic neurons are found in the BF and pontomesencephalic (PM) areas as projection neurons, as well as in the CPu as interneurons, where they play a significant role in motivational and neurocognitive functions^30^. To gain insight into the mechanisms underlying behavioral deficits, we first measured the protein expression levels of ChAT responsible for the synthesis of acetylcholine (Ach) and found that it was downregulated in the BF and PFC (one of the main targets for projections of BF cholinergic neurons and that is closely linked to schizophrenia), but not in the CPu nor PM areas (Fig. 3a and Supplementary Fig. 2b, f) in *ChAT-Cre;Hrh1*^*fl/fl*^ mice. The distinctive ChAT expression in different brain regions following H_1_R deficiency may be due to the diverse morphological and functional properties of cholinergic neurons. Using HPLC-MS/MS, we also observed decreased Ach concentration in the BF, but not in the CPu (Fig. 3b). Furthermore, the extracellular Ach level in the medial prefrontal cortex (mPFC) was reduced by employing a microdialysis component in combination with HPLC-MS/MS, thus suggesting a decreased release of Ach in the mPFC from BF cholinergic neurons (Fig. 3c). However, the deficit of Ach here may not be due to quantitative alterations of cholinergic neurons, since the number of cholinergic neurons in BF was unchanged after the loss of histamine H_1_R (Supplementary Fig. 2c). Further, the excitability of cholinergic neurons in the nucleus of the horizontal limb of the diagonal band (HDB) or NBM areas of BF was examined by patch clamp techniques. The threshold current to elicit action potential in HDB or NBM cholinergic neurons from mutant mice was higher than that in controls, while the spike numbers were remarkably lower than that in controls with the increase of the injected currents, suggesting that the deletion of H_1_R in cholinergic neurons reduces the excitability of cholinergic neurons in the HDB and NBM areas of BF (Fig. 3d, e). In addition, ISH and immunohistochemistry were performed following patch clamp recordings to simultaneously observe the alteration in excitability of HDB cholinergic neurons and their *Hrh1* mRNA expression (Supplementary Fig. 9). We found that cholinergic neurons expressing high level of *Hrh1* mRNA in *Hrh1*^*fl/fl*^ control mice showed high intrinsic excitability, while cholinergic neurons expressing absent or low *Hrh1* mRNA in *ChAT-Cre;Hrh1*^*fl/fl*^ mice showed low intrinsic excitability. These observations suggest that H_1_R knockout alters function of cholinergic cells in the BF but not in the CPu, and thus that dysfunction of BF cholinergic cells may underlie the observed behavioral abnormalities. Moreover, the reduced expression of ChAT and excitability of cholinergic neurons in the BF can be rescued by a chronic treatment of risperidone, while risperidone had no effect on *Hrh1*^*fl/fl*^ control mice (Fig. 3h, i).

An imbalance between excitation and inhibition in the PFC is a key feature of neural circuits in schizophrenia and likely critical for most of the characteristic symptoms^31^. Postmortem studies examining the brains of schizophrenic patients suggest that inhibitory inputs onto excitatory neurons are reduced in schizophrenia^32^. It has been reported that both superficial and deep layers in the mPFC receive the cholinergic projections from the BF, effecting both glutamatergic and GABAergic neuronal excitability^33,34^, so the excitation and inhibition balance was tested in either layer 2/3 or layer 5 of prelimbic and infralimb areas. Spontaneous excitatory postsynaptic current (sEPSC) and spontaneous inhibitory postsynaptic current (sIPSC) were recorded by alternately clamping at −60 and +10 mV, respectively, to directly compare glutamatergic and GABAergic synapses on a cell-by-cell basis^35^. This strategy revealed that the sEPSC charge transfer of mPFC layer 2/3 pyramidal cells was unchanged while the sIPSC charge transfer was robustly decreased in *ChAT-Cre;Hrh1*^*fl/fl*^ mice, leading to an increased sEPSC/sIPSC charge transfer ratio (Fig. 3f), which can also be reversed by a treatment of risperidone (Fig. 3j). The decrease of sIPSC and increase of sEPSC/sIPSC charge transfer ratio was also observed in pyramidal cells in layer 5, although the alteration was less prominent compared with that in layer 2/3 (Fig. 3g). These results indicate that deletion of H_1_R in cholinergic neurons selectively induces dysfunction of cholinergic neurons in the BF, a subsequent decrease in Ach release, and excitation/inhibition imbalance in the mPFC, which could be the underlying mechanism for the behavioral deficits observed here.

### Re-expression of histamine H_1_R in the BF cholinergic neurons rescues behavioral deficits manifested in *ChAT-Cre;Hrh1*^*fl/fl*^ mice

**Fig. 4:**
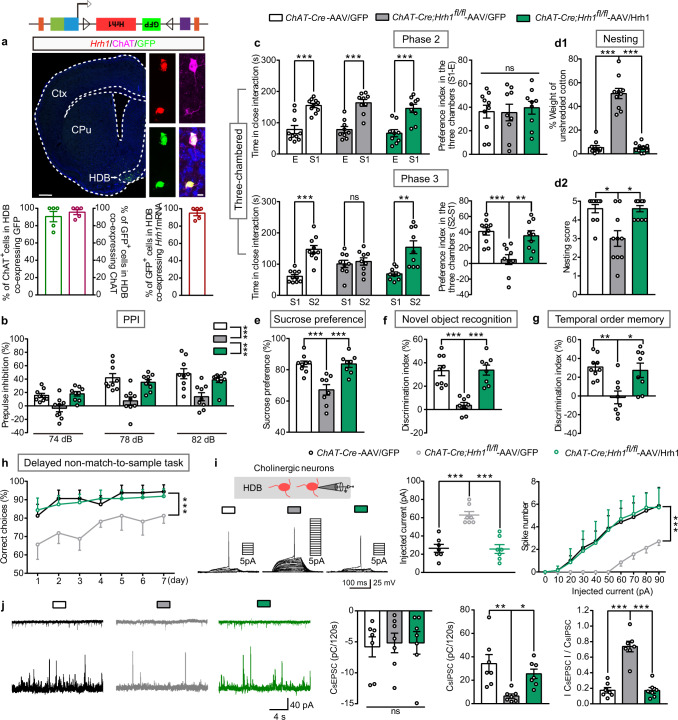
Re-expression of histamine H_1_R in cholinergic neurons in the HDB of BF rescues behavioral deficits manifested in *ChAT-Cre;Hrh1*^*fl/fl*^ mice. **a** Representative images of H_1_R-GFP expression in the HDB of BF in *ChAT-Cre;Hrh1*^*fl/fl*^ mice after the microinjection of AAV-CAG-FLEX-*Hrh1*-GFP (AAV/Hrh1), enlarged images of RNAscope in situ hybridization of *Hrh1* and *GFP* mRNA, and immunostaining of ChAT. The percentage of ChAT^+^ cholinergic neurons co-expressing GFP, percentage of GFP^+^ cells co-expressing ChAT, and percentage of GFP^+^ cells co-expressing *Hrh1* mRNA in the HDB were quantified. Left: scale bar, 500 μm. Right: scale bar, 10 μm. *n* = 5 mice. **b**–**h** Sensorimotor gating, social behavior, hedonic function, and cognitive behavior in control *ChAT-Cre* mice injected with AAV-CAG-FLEX-GFP (AAV/GFP), and *ChAT-Cre;Hrh1*^*fl/fl*^ mice either injected with AAV/GFP or AAV/Hrh1. **b** Percentage of prepulse inhibition of the auditory startle reflex across different prepulse intensities. **c** Time in close interaction and preference index during sociability testing (phase 2) when exposed to a stranger mouse S1. Time in close interaction and preference index during subsequent social novelty recognition testing (phase 3) when exposed to a new stranger mouse S2 together with S1. **d** The percentage of the weight of cotton left unshredded and nesting score in nest-building test. **e** Test for sucrose preference as a percentage of all fluid intake within a 48 h period. **f** Discrimination index in novel object recognition test. **g** Discrimination index in temporal order memory test. **h** Percentage of correct choices during choice runs in delayed non-match-to-sample task. **i** Threshold current to elicit action potential with the increase of injected currents in HDB of BF cholinergic neurons recorded by whole-cell patch-clamp. Spike numbers with the increase of injected currents in BF cholinergic neurons recorded by whole-cell patch-clamp. **j** Representative traces showing sEPSC (upper) and sIPSC (lower) recorded at −60 mV (upper) and +10 mV (lower) in the same mPFC layer 2/3 pyramidal cell from *ChAT-Cre*-AAV/GFP (black), *ChAT-Cre;Hrh1*^*fl/fl*^-AAV/GFP (gray), or *ChAT-Cre;Hrh1*^*fl/fl*^-AAV/Hrh1 (green). Quantification of sEPSC and sIPSC charge transfer and sEPSC/sIPSC charge transfer ratios in *ChAT-Cre*-AAV/GFP, *ChAT-Cre;Hrh1*^*fl/fl*^-AAV/GFP, or *ChAT-Cre;Hrh1*^*fl/fl*^-AAV/Hrh1 mice. All data are presented as mean ± s.e.m. and error bars represent s.e.m. *P ≤ 0.05, **P ≤ 0.01, ***P ≤ 0.001, ns nonsignificant. See also Supplementary Data 2 for further statistical information. Source data are provided as a Source Data file.

To further verify H_1_R mutation in the BF cholinergic neurons is critical for inducing behavioral deficits in *ChAT-Cre;Hrh1*^*fl/fl*^ mice, a Cre-dependent adeno-associated virus containing floxed *Hrh1*-GFP (AAV-CAG-FLEX-*Hrh1*-GFP) was employed to rescue the H_1_R expression in the BF. It has been reported that both the cholinergic neurons in the NBM and HDB of BF have projections to the mPFC, while the cholinergic neurons in HDB have more of such projections^33,34^. So, we bilaterally injected AAV-CAG-FLEX-*Hrh1*-GFP into either the NBM or HDB area in the BF of *ChAT-Cre;Hrh1*^*fl/fl*^ mice to selectively re-express histamine H_1_R in cholinergic neurons (Fig. 4a and Supplementary Fig. 10a). 95.52% in the HDB and 96.14% in the NBM of GFP-labeled neurons stained positively for ChAT, and 90.44% in the HDB and 90.92% in the NBM of ChAT-positive neurons were infected with AAV-CAG-FLEX-*Hrh1*-GFP. Consistent with the behavioral findings in Fig. 2, *ChAT-Cre;Hrh1*^*fl/fl*^ mice injected with control virus expressing only GFP (AAV-CAG-FLEX-GFP) showed disrupted PPI, social impairments, anhedonia-like behavior, and cognitive impairments. However, re-expression of H_1_R in either the HDB or NBM area in the BF completely rescued all of the altered behaviors (Fig. 4b–h and Supplementary Fig. 10b–g). These findings suggest that such behavioral phenotypes are not due to a developmental disorder but instead result from H_1_R dysfunction in BF cholinergic neurons. In contrast, H_1_R re-expression in the CPu cholinergic neurons had no effect on disrupted PPI, social impairments, anhedonia-like behavior, and cognitive impairments in *ChAT-Cre;Hrh1*^*fl/fl*^ mice (Supplementary Fig. 11). Therefore, these data indicate that H_1_R in the BF rather than in the CPu cholinergic neurons was specifically involved in the behavioral deficits. Moreover, the excitability of cholinergic neurons in the HDB of BF and E/I balance in layer 2/3 of prelimbic and infralimb areas recovered when H_1_R was re-expressed in these cells in *ChAT-Cre;Hrh1*^*fl/fl*^ mice (Fig. 4i, j). Taken together, these data provide solid evidence that loss of function of histamine H_1_R in BF cholinergic neurons is pivotal in the formation of the behavioral deficits seen in *ChAT-Cre;Hrh1*^*fl/fl*^ mice.

### Chemogenetic activation of cholinergic neurons in the BF rescues behavioral deficits in *ChAT-Cre;Hrh1*^*fl/fl*^ mice

**Fig. 5:**
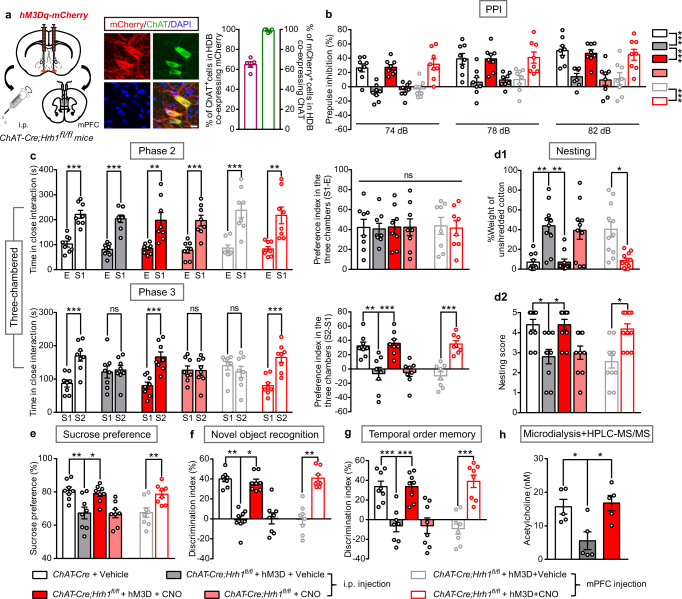
Chemogenetic activation of cholinergic neurons in the HDB of BF rescues behavioral deficits in *ChAT-Cre;Hrh1*^*fl/fl*^ mice. **a** Schematic diagram of microinjection of AAV-EF1α-DIO-hM3Dq-mCherry (hM3D-mCherry) in the HDB of BF in *ChAT-Cre;Hrh1*^*fl/fl*^ mice, and representative images of hM3Dq-mCherry expression in HDB ChAT^+^ cholinergic neurons. The percentage of ChAT^+^ cholinergic cells co-expressing mCherry and percentage of mCherry expressed cells co-labeling ChAT were quantified. Scale bar, 10 μm, *n* = 5 mice. **b**–**g** Sensorimotor gating, social behavior, hedonic function, and cognitive behavior in *ChAT-Cre;Hrh1*^*fl/fl*^ mice either injected with hM3Dq-mCherry or not. CNO was administrated either by i.p. injection or by intra-mPFC microinjection. **b** Percentage of prepulse inhibition of the auditory startle reflex across different prepulse intensities. **c** Time in close interaction and preference index during sociability testing (phase2), when exposed to a stranger mouse S1. Time in close interaction and preference index during subsequent social novelty recognition testing (phase 3), when exposed to a new stranger mouse S2 together with S1. **d** The percentage of the weight of unshredded cotton and nesting score in nest-building test. **e** Test for sucrose preference as a percentage of all fluid intake within a 48 h period. **f** Discrimination index in novel object recognition test. **g** Discrimination index in temporal order memory test. **h** Extracellular acetycholine level in the mPFC collected by microdialysis and examined by HPLC-MS/MS. All data are presented as mean ± s.e.m. and error bars represent s.e.m. *P ≤ 0.05, **P ≤ 0.01, ***P ≤ 0.001, ns nonsignificant. See also Supplementary Data 2 for further statistical information. Source data are provided as a Source Data file.

To further confirm the involvement of the dysfunction of BF cholinergic neurons induced by the deletion of H_1_R in the behavioral phenotypes in *ChAT-Cre;Hrh1*^*fl/fl*^ mice, we bilaterally injected a Cre-dependent adeno-associated virus (AAV-EF1α-DIO-hM3Dq-mCherry) into the BF (HDB or NBM areas) of *ChAT-Cre;Hrh1*^*fl/fl*^ mice. Immunohistochemical staining showed 98.85% in the HDB or 94.64% in the NBM of mCherry-labeled neurons were ChAT^+^ neurons (Fig. 5a and Supplementary Fig. 12a), indicating that hM3Dq was primarily restricted to the BF cholinergic neurons. When clozapine N-oxide (CNO, 1 mg/kg) was intraperitoneally administered to activate BF cholinergic neurons, behavioral abnormalities were completely ameliorated in the *ChAT-Cre;Hrh1*^*fl/fl*^ mice, including disrupted PPI, social impairments, anhedonia-like behavior, and cognitive impairments (Fig. 5b–g). This action is not related to the direct action of CNO, since the treatment of CNO in *ChAT-Cre;Hrh1*^*fl/fl*^ mice lacking expression of hM3Dq had no effect on their behavioral abnormalities. Furthermore, intracranial microinjection of CNO (3 μM, 100 nL) in the mPFC completely reversed the altered behaviors of the *ChAT-Cre;Hrh1*^*fl/fl*^ mice expressing hM3Dq either in the HDB or NBM (Fig. 5b–g and Supplementary Fig. 12b–g). Ach release in PFC was also tested in *ChAT-Cre;Hrh1*^*fl/fl*^ mice expressing hM3Dq in the HDB (Fig. 5h). We found that activation of HDB cholinergic neurons completely restored extracellular ACh level in PFC that is consistent with their action on behavioral deficits in *ChAT-Cre;Hrh1*^*fl/fl*^ mice. These results indicate that the dysfunction of BF cholinergic neurons, in particular with their projections to the mPFC, contributes to sensorimotor gating ability deficit, social impairments, and anhedonia-like behavior induced by the deletion of H_1_R in cholinergic neurons.

### Chemogenetic inhibition of cholinergic neurons in the BF induces sensorimotor gating ability deficit, social impairments and anhedonia-like behavior, as well as increased susceptibility to MK-801 induced hyperlocomotion

**Fig. 6:**
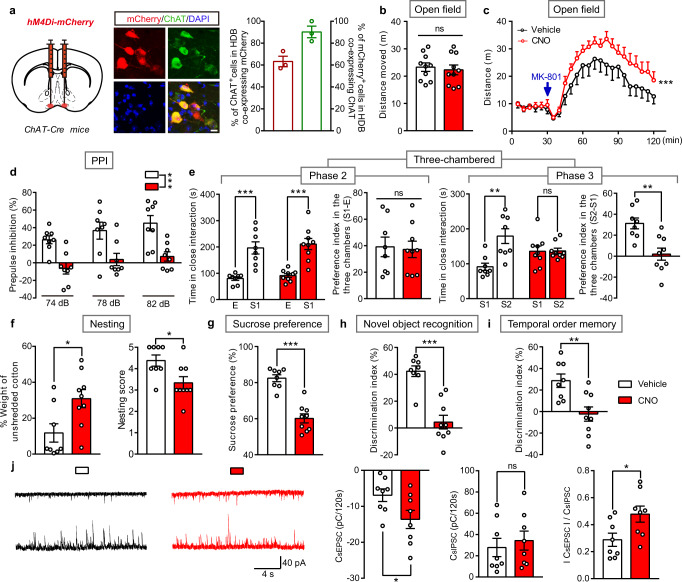
Chemogenetic inhibition of cholinergic neurons in the BF induces sensorimotor gating ability deficit, social impairments, anhedonia-like behavior, and cognitive impairments, together with increased susceptibility to MK-801 induced hyperlocomotion. **a** Schematic diagram of microinjection of AAV-EF1α-DIO-hM4Di-mCherry (hM4Di-mCherry) in the HDB of BF in *ChAT-Cre* mice, and representative images of hM4Di-mCherry expression in HDB ChAT^+^ cholinergic neurons. The percentage of ChAT^+^ cholinergic cells co-expressing mCherry and percentage of mCherry expressed in cells co-labeling ChAT were quantified. Scale bar, 10 μm. *n* = 3 mice. **b**–**i** Locomotion, sensorimotor gating, social behavior, hedonic function, and cognitive behavior in *ChAT-Cre* mice injected with hM4Di-mCherry in the HDB and treated with CNO or vehicle. **b** The cumulative distance traveled in the open field for 15 min in locomotion test. **c** The distance traveled in the open field for every 5 min was quantified and MK-801 was administrated 30 min after the free exploration phase. **d** Percentage of prepulse inhibition of the auditory startle reflex across different prepulse intensities. **e** Time in close interaction and preference index during sociability testing (phase 2), when exposed to a stranger mouse S1. Time in close interaction and preference index during subsequent social novelty recognition testing (phase 3), when exposed to a new stranger mouse S2 together with S1. **f** The percentage of the weight of unshredded cotton and nesting score in nest-building test. **g** Test for sucrose preference as a percentage of all fluid intake within a 48 h period. **h** Discrimination index in novel object recognition test. **i** Discrimination index in temporal order memory test. **j** Representative traces showing sEPSC (upper) and sIPSC (lower) recorded at –60 mV (upper) and +10 mV (lower) in the same mPFC layer 2/3 pyramidal cell from Vehicle (black) or CNO (red) mice. Quantification of sEPSC and sIPSC charge transfer and sEPSC/sIPSC charge transfer ratios in *ChAT-Cre* mice treated with CNO or vehicle. All data are presented as mean ± s.e.m. and error bars represent s.e.m. *P ≤ 0.05, **P ≤ 0.01, ***P ≤ 0.001, ns nonsignificant. See also Supplementary Data 2 for further statistical information. Source data are provided as a Source Data file.

We hypothesized that direct chemogenetic inhibition of cholinergic neurons in the BF would produce a similar phenotype as blocking its expression of H_1_R. Bilateral virus injections (AAV-EF1α-DIO-hM4Di-mCherry) were delivered into the BF of *ChAT-Cre* mice, resulting in expression of the inhibitory hM4Di designer receptor in cholinergic neurons, which are activated by CNO (Fig. 6a). When CNO (i.p. 1 mg/kg) was administrated for 1 week to reduce BF cholinergic activity, *ChAT-Cre* mice expressing hM4Di showed disrupted PPI, social impairments, anhedonia-like behavior, and cognitive impairments, but no change in locomotion, which is similar to behavioral symptoms manifested in *ChAT-Cre;Hrh1*^*fl/fl*^ mice (Fig. 6b, d–i). Notably, although MK-801 can elicit hyperactive locomotion in the open field by expressing hM4Di in *ChAT-Cre* mice no matter they are injected with CNO or not, the mice show higher sensitivity to MK-801 after injection with CNO (Fig. 6c). Together, these data suggest that chemogenetic inhibition of cholinergic neurons in the BF increases susceptibility to MK-801 induced hyperlocomotion in addition to causing behavioral deficits observed in *ChAT-Cre;Hrh1*^*fl/fl*^ mice (Supplementary Fig. 5). Although the sEPSC/sIPSC charge transfer ratio in mPFC layer 2/3 pyramidal cells was also increased after chemogenetic inhibition of cholinergic neurons as that in *ChAT-Cre;Hrh1*^*fl/fl*^ mice (Fig. 3f), the sEPSC charge transfer was increased while the sIPSC charge transfer was unchanged (Fig. 6j). The hyperlocomotion has been found to be related to mPFC excitation/inhibition imbalance^31,36,37^. The different behavioral feature in locomotion in *ChAT-Cre;Hrh1*^*fl/fl*^ mice and mice having chemogenetic inhibition of cholinergic neurons may be due to different pattern of alterations in excitatory and inhibitory transmission. Our findings further support the proposal that histamine H_1_R-dependent inhibition of BF cholinergic neurons, but not direct inhibition of cholinergic neurons, may be specific to the behavioral deficits, involving sensorimotor gating ability deficit, social impairments, anhedonia-like behavior, and cognitive impairment.

### Over-expression of histamine H_1_R in BF cholinergic neurons selectively improves sensorimotor gating ability deficit, social impairments, and anhedonia-like behavior but not the hyperlocomotion

**Fig. 7:**
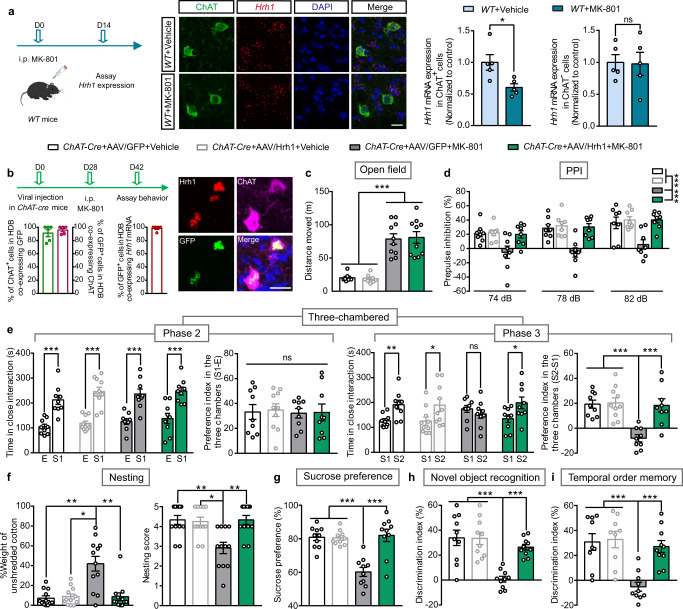
Over-expression of histamine H_1_R in BF cholinergic neurons selectively rescues sensorimotor gating ability deficit, social impairments, anhedonia-like behavior, and cognitive impairments, but not hyperlocomotion after MK-801 exposure. **a** Schedule of 2-week MK-801 injections and then *Hrh1* mRNA expression assay by RNAscope in situ hybridization in *WT* mice. Representative images of *Hrh1* mRNA and choline acetyltransferase (ChAT) expression in the HDB of BF after MK-801 or vehicle administration. The *Hrh1* mRNA in the ChAT^+^ or ChAT^-^ cells were quantified. Scale bar, 20 μm. **b** Schedule of microinjection of AAV-CAG-FLEX-*Hrh1*-GFP (AAV/Hrh1) in *ChAT-Cre* mice, MK-801 administration and behavior assay. Representative images of *Hrh1-GFP* expression in the HDB of BF in *ChAT-Cre* mice after the microinjection of AAV/Hrh1, enlarged image of in situ hybridization of *Hrh1* and *GFP* mRNA, and immunostaining of ChAT. The percentage of ChAT^+^ cholinergic neurons co-expressing GFP^+^, percentage of GFP^+^ cells co-expressing ChAT, and percentage of GFP^+^ cells co-expressing *Hrh1* mRNA in the BF were quantified. Scale bar, 20 μm, *n* = 5 mice. **c**–**i** Locomotion, sensorimotor gating, social behavior, hedonic function, and cognitive behavior in control *ChAT-Cre* mice injected with AAV/Hrh1 and AAV-CAG-FLEX-GFP (AAV/GFP) either injected with vehicle or MK-801. **c** The cumulative distance traveled in open field for 15 min in locomotion test. **d** Percentage of prepulse inhibition of the auditory startle reflex across different prepulse intensities. **e** Time in close interaction and preference index during sociability testing (phase 2), when exposed to a stranger mouse S1. Time in close interaction and preference index during subsequent social novelty recognition testing (phase 3), when exposed to a new stranger mouse S2 together with S1. **f** The percentage of the weight of unshredded cotton and nesting score in nest-building test. **g** Test for sucrose preference as a percentage of all fluid intake within a 48 h period. **h** Discrimination index in novel object recognition test. **i** Discrimination index in temporal order memory test. All data are presented as mean ± s.e.m. and error bars represent s.e.m. *P ≤ 0.05, **P ≤ 0.01, ***P ≤ 0.001, ns nonsignificant. See also Supplementary Data 2 for further statistical information. Source data are provided as a Source Data file.

Mice chronically treated with NMDA receptor antagonists, such as MK-801, represent one of the most widely used pharmacological models for schizophrenia^38^. We found that the *Hrh1* mRNA expression was decreased in the HDB of BF cholinergic neurons after the injections of MK-801 for 2 weeks, suggesting H_1_R is involved in the pathogenesis of MK-801 induced behavioral deficits (Fig. 7a). To further demonstrate whether H_1_R in cholinergic neurons is associated with the behavioral deficits in an experimental schizophrenia model, and whether H_1_R is an appropriate therapeutic target, *ChAT-Cre* mice were injected with AAV-CAG-FLEX-*Hrh1*-GFP into the BF to overexpress H_1_R in BF cholinergic neurons, followed by treatment with MK-801 for 2 weeks (0.2 mg/kg/d) (Fig. 7b). RNAscope ISH of H_1_R mRNA showed successful over-expression of H_1_R in the BF cholinergic neurons (Fig. 7b). We found that over-expression of H_1_R in BF cholinergic neurons had no effect on locomotor activity, but did restore full functional capacity for the disrupted PPI, social impairments, anhedonia-like behavior, and cognitive dysfunction after MK-801 administration (Fig. 7c–i).

## Discussion

In the present study, we revealed that the *Hrh1* mRNA expression in cholinergic neurons was only reduced in patients with schizophrenia having both positive and negative symptoms, but not the patients with schizophrenia having positive symptoms only. Moreover, the deficiency of H_1_R in cholinergic neurons trigger behavioral and pathophysiological features that may be associated with negative symptoms found in schizophrenia. In particular, the *ChAT-Cre;Hrh1*^*fl/fl*^ mice exhibited deficits in social novelty recognition and nesting, as well as a reduced preference for sweet solution, which are linked to the social impairments and anhedonia in the negative symptoms of schizophrenia^39–42^. Importantly, impaired sensorimotor gating which were often observed in patients with schizophrenia, was found in *ChAT-Cre;Hrh1*^*fl/fl*^ mice as indicated by decreased PPI of the startle reflex. The deficits in sensorimotor gating can be rescued by both the typical and atypical antipsychotics, while the deficits in social interaction were only rescued by the atypical antipsychotics, which are consistent to the actions of antipsychotics on patients with schizophrenia in clinic^43,44^. In addition, the *ChAT-Cre;Hrh1*^*fl/fl*^ mice had cognitive symptoms such as deficits in working memory, recognition memory, and temporal memory, that are also the cardinal disturbances in patients with schizophrenia. However, psychomotor agitation in locomotion, which is linked to positive symptom, was not observed even after the administration of psychostimulant drug. The excitation/inhibition imbalance in the mPFC of *ChAT-Cre;Hrh1*^*fl/fl*^ mice, that can also be relieved by risperidone, is consistent with the pathophysiological features in the schizophrenia^31,45,46^. In addition, over-expression of H_1_R in cholinergic neurons rescued all the behavioral deficits related to negative symptoms and cognitive impairments, but not the hyperactive locomotion, in an animal model of schizophrenia induced by repeated MK-801. Together, our results provided strong evidence to demonstrate that a deficiency of H_1_R in cholinergic neurons selectively elicits behavioral deficits which may be related to negative symptoms of schizophrenia.

We found that the ChAT expression and Ach concentration were reduced in the BF except the CPu in *ChAT-Cre;Hrh1*^*fl/fl*^ mice. The behavioral deficits in *ChAT-Cre;Hrh1*^*fl/fl*^ mice can be attenuated by re-expression of H_1_R in cholinergic neurons in the BF, but not that in CPu. Our results suggested H_1_R in cholinergic neurons plays distinctive roles at different brain regions and BF specific loss of H_1_R may be critical for the pathogenesis of sensorimotor gating ability deficit, social impairments, anhedonia, and cognitive impairments. Indeed, the dysfunction of BF cholinergic neurons has been detected in *ChAT-Cre;Hrh1*^*fl/fl*^ mice. The activation or inhibition of BF cholinergic neurons by using chemogenetic approaches can relieve or provoke behavioral deficits. Meanwhile, H_1_R in dopaminergic or glutamatergic neurons seems not related the schizophrenia, since neither *DAT-Cre;Hrh1*^*fl/fl*^ nor *CaMKIIα-Cre;Hrh1*^*fl/fl*^ mice displayed behavioral deficits. Our results thus implicate cell-type specific and BF-region specific loss of H_1_R functionality is a cellular and neural circuit mechanism in the pathogenesis of sensorimotor gating ability deficit, social impairments, and anhedonia. Historically, the basis for negative symptoms in schizophrenia has been attributed to downregulation of NMDA receptor-related glutamatergic transmission in the PFC that results in a lack of tonic excitation at the dopamine neuron and then insufficient dopamine reaching the cortex^4,47^. Our study implies that dysfunction of H_1_R in cholinergic neurons may also contribute to the pathogenesis of negative symptoms in schizophrenia. Moreover, to the best of our knowledge, there are no genuine animal models for the negative symptoms of schizophrenia^31^. Although models have been created for the studies of schizophrenia related to genetic risk factors and pathophysiological mechanisms, such as the mutated gene *Disrupted in schizophrenia-1*, *DISC1*, the microdeletion on region q11.2 of chromosome 22, and blocking of NMDA receptors by MK-801, these models are also associated with other disorders such as bipolar disorder or autism, and involve both positive and negative symptoms^20,48,49^. Our finding suggests that the *ChAT-Cre;Hrh1*^*fl/fl*^ mice may be used for the development and screening of drugs specifically aimed at patients with predominantly negative symptoms.

Studies examining the brains of patient postmortem or animal models of schizophrenia both suggest that excitation/inhibition imbalance of the PFC is the endophenotype of schizophrenia^31,50,51^. This imbalance may be produced by reduced inhibitory inputs onto excitatory neurons or concurrently with either an increase or a decrease in excitatory inputs^31,52^. Cholinergic function has been found to elevate the cortical signal-to-noise ratio by increasing the excitability and firing rates of GABAergic interneurons or by directly acting on pyramidal neurons in attention, cue detection, and cognitive processing. However, the disruption of such modulation may disturb excitation/inhibition balance and reduce the signal-to-noise ratio in cortical networks that contribute to schizophrenia^53–55^. H_1_R has been found to modulate cholinergic activity by regulation of its depolarization^56^. Here, the dysfunction of H_1_R in cholinergic neurons induces hypofunction of cholinergic projections from the BF to the PFC, thus eliciting an excitation/inhibition imbalance in the PFC, which could be upstream of the glutamate-dopamine disorder in conventional theories or could function in parallel causal processes. Actually, the expression of H_1_R was decreased in the BF of patients with schizophrenia having negative symptoms along with the reduced ChAT expression, further supporting that the aberrant BF cholinergic neurons resulted from H_1_R dysfunction is closely linked to the negative symptoms of schizophrenia.

Notably, the phenotype of *ChAT-Cre;Hrh1*^*fl/fl*^ mice is ostensibly different from direct inhibition of cholinergic neurons by chemogenetic techniques that use hM4Di. In these experiments, an increased susceptibility to hyperactive locomotion is triggered in addition to the behavioral deficits related to negative symptoms. Although the mPFC excitation/inhibition balance was also increased after chemogenetic inhibition of cholinergic neurons, the excitatory and inhibitory transmission displayed different pattern of alterations compared with that in *ChAT-Cre;Hrh1*^*fl/fl*^ mice. Based on previous studies suggesting mPFC excitation/inhibition imbalance is the endophenotype of schizophrenia^31^, we propose that the decrease in inhibitory transmission may be a hint for the pathogenesis of negative symptom in patients with schizophrenia. Cholinergic receptor-related drugs exhibit antipsychotic effects both on positive and negative symptoms^57–59^. Moreover, risperidone may directly recover the function of BF cholinergic neurons, rather than acting on the H_1_R, to correct the mPFC imbalance, which could contribute to its effect on both the positive and negative symptoms in schizophrenia. However, over-expression of H_1_R in cholinergic neurons only completely rescued the behavioral deficits including sensorimotor gating ability deficit, social impairments, anhedonia-like behavior, and cognitive impairments, but not the hyperlocomotion, in the MK-801-induced schizophrenia model (Fig. 7). These lines of evidence further support the proposal that H_1_R-dependent dysfunction of cholinergic neurons from the BF to the PFC is the potential etiology and a logical therapeutic target for negative symptoms of schizophrenia. Clinical therapy for negative symptoms of schizophrenia would benefit from further study to develop a drug carrier for an H_1_R-targeted drug specifically delivered to cholinergic neurons of the BF, while avoiding non-selective direct modulation of cholinergic neurons. Moreover, the degeneration of cholinergic neurons in the BF, including NBM area, has been observed in Alzheimer’s disease patients with either mild cognitive decline or severe cognitive decline, exhibiting decreased number of cholinergic neurons and ChAT expression together with the accumulation of hyperphosphorylated tau and neuronal fibrillary tangles^60^. It suggests that the cholinergic neurons in the BF are very vulnerable, however, the dysfunction or degeneration of cholinergic neurons into different profile may provoke different pathological processes. Nevertheless, H_1_R dysfunction may be a trigger for BF cholinergic neurons to step in a pathological progress for negative symptom in schizophrenia.

In conclusion, our results demonstrate that dysfunction of H_1_R expression in the BF cholinergic neurons, but not in the glutamatergic or dopaminergic neurons, is crucial for the sensorimotor gating ability deficit, social impairments, anhedonia-like behavior, and cognitive impairments. This finding may greatly enrich the conventional theory regarding the genetic and biochemical bases of negative symptoms in schizophrenia and provide a selective therapeutic target. In addition, *ChAT-Cre;Hrh1*^*fl/fl*^ mice might be used for pathophysiological studies and drug screens for patients suffering from the negative symptoms of schizophrenia.

## Methods

### Animals

*Hrh1*^*fl/fl*^ mice were commercially generated by standard homologous recombination at the Nanjing Biomedical Research Institute of Nanjing University, Nanjing, China. Exon 3, encoding the core region of *Hrh1*, was flanked on either side by loxP sequences. To specifically delete *Hrh1* in DAT^+^ dopaminergic neurons, CaMKIIα^+^ glutamatergic neurons, and ChAT^+^ cholinergic neurons, *Hrh1*^*fl/fl*^ homozygous mice were mated with *DAT-Cre* mice (Jax No. 006660), *CaMKIIα-Cre* mice (Jax No. 005359), or *ChAT-Cre* mice (Jax No. 006410), respectively. To determine Cre distribution, we crossed *ChAT-Cre* mice with *Ai14* mice, which are used as a Cre reporter strain (Jax No. 007914). All mice were bred onto a C57BL/6 J genetic background. The environmental conditions in the mouse facility were: 12 h light and 12 h dark cycle (light on from 8:00 a.m. to 8:00 p.m.), temperature range of 21–23 °C, humidity range of 40–50%, and free access to food and water. All behavior experiments were performed each day between 10:00 a.m. and 7:00 p.m. The use and care of the mice were in accordance with the guidelines of the Animal Advisory Committee of Zhejiang University and the US National Institutes of Health Guidelines for the Care and Use of Laboratory Animals. All procedures were approved by the Animal Advisory Committee of Zhejiang University.

For genotyping of *Hrh1*^*fl/fl*^ mice, two pairs of oligos were used (Supplementary Fig. 1b). The primers are listed in Supplementary Data 3. For the pair of Hrh1-loxptF and Hrh1-loxptR, the PCR products were 446 (floxed allele) and 327 bp (wild-type allele). For the pair of Hrh1-loxptF and Hrh1-FRT-tR, the PCR products were 354 bp (deleted allele). The program included 95 °C for 5 min (1×); 95 °C for 30 s, 58 °C for 30 s, and 72 °C for 30 s (40×); and 72 °C for 5 min (1×).

### Post-mortem brain material

For this study, human post-mortem brain tissue was acquired from the Netherlands Brain Bank (NBB). Informed consent was obtained from all the participants according to the Declaration of Helsinki and the use of the material and clinical information for research purposes had been obtained by the NBB. We included tissues from 14 patients with schizophrenia and 12 controls (schizophrenia: 7 females, 7 males; controls: 7 females and 5 males). The diagnosis of schizophrenia was made during life and confirmed according to the Diagnostic and Statistical Manual of Mental Disorders IV by qualified psychiatrists using the extensive medical records of the NBB, which also contained well-documented diagnoses and onset of schizophrenia from psychiatric clinics. Exclusion criteria were any other neurological or psychiatric diseases. Eleven patients with schizophrenia (Number 1–11) displayed both positive and negative symptoms, including withdrawn, depressed, passive, or inactive characteristics, but three patients with schizophrenia (Number 12–14) only had positive symptoms without apparent negative symptoms. Controls had to be non-demented and had no known history of a psychiatric disorder. The patients with schizophrenia and controls were matched for sex, age (schizophrenia: Mean = 66.8, SEM = 3.04; controls: Mean = 69.1, SEM = 3.1, and *p* = 0.62), postmortem delay (PMD), month of death, CSF pH, brain weight, and Braak stage of Alzheimer pathology. All study protocols were complied with the guidelines for the conduct of research involving human subjects as established by the Ethics Committee of Zhejiang University School of Medicine. All procedures were approved by the Ethics Committee of Zhejiang University School of Medicine. Detailed clinico-pathological information and *P* values of the matched parameter are given in Supplementary Data 1.

### Behavior assays

Mice at 8–14 weeks of age were used for all tests. They were habituated in the testing room for at least 30 min before the test. All behavioral tasks were performed during the light phase. All behavioral assays were carried out with researchers blinded to the genotype.

### Olfactory Habituation/Dishabituation test

Each mouse was introduced to a clean mouse cage containing fresh bedding for 30 min before the experiment. Each cotton swab with odor cue was delivered through a hole in the center of the cage top positioned 7 cm above the bedding. The olfactory cues were designed to present familiar and unfamiliar odors, including (I) tap water, (II) coffee, (III) urine of a familiar mouse of the same strain and sex, and (IV) urine of another unfamiliar mouse of the same strain and sex. The time spent sniffing the swab was quantitated with a stopwatch by an observer uninformed about the genotype of the subject mouse. Sniffing was scored when the mouse’s nose was within 2 cm of the cotton swab. Each swab was presented for a 2-min period and inter-session interval was 1 min.

### Accelerating rotarod

Mice were tested concurrently in five separated 6 cm-wide compartments on a rod ~3 cm in diameter and elevated 35 cm by using an accelerating rotarod (YLS-4C). The apparatus started at an initial speed of 5 rpm and gradually accelerated to 40 rpm. The latency in time to fall from the rod was recorded with a cut-off time of 5 min. Each animal was assessed over eight trials with 20-min inter-trial intervals.

### Hot plate test

Mice were placed on the hot plate (55 ± 0.1 °C). The mice were constrained to the hot plate by clear acrylic walls (19 cm tall, open top). The latency in time to respond with either hind paw lick, hind paw flick, or jump was recorded and the mouse was immediately removed from the hot plate and returned to its home cage.

### Repetitive behaviors

After a 30 min habituation period, mice in their home cages with fresh bedding were observed for measurement of the time spent in repetitive behaviors including digging, grooming, and jumping during 10 min. Digging behavior was defined as the behavior of a mouse where it coordinately uses two fore legs or hind legs to dig out or displace bedding materials. Grooming behavior was defined as stroking or scratching of face, head, or body with the two forelimbs, or licking body parts. Jumping was defined as the behavior of a mouse where it rears on its hind legs at the corner of the cage, or along the side walls, and jumps so that the two hind legs are simultaneously off the ground.

### Prepulse inhibition (PPI)

PPI test was conducted in SR-LAB startle chambers. After the mouse was placed in the test chamber, the sessions began with a 5 min acclimation interval to a background white noise of 70 dB throughout the session, which was followed by a block of five 20 ms startle pulses of 120 dB. Subjects then received six blocks to measure PPI. Each block of trials consisted of eight different trial types presented pseudo-randomly across blocks: startle tone only, no stimuli, pulse tone only (74, 78, or 82 dB), and pulse paired with startle tone, followed later by a block of five 20 ms startle pulses of 120 dB. The maximum amplitude of startle response was recorded for every trial. The average value for every type of trial across six blocks was used for the statistical analysis. Percent PPI was calculated using the following formula: [1 - (averaged startle response to prepulse before startle stimulus/averaged response to startle stimulus)] × 100.

### Open-field test

A clear plexiglas box (45 × 45 × 45 cm) was used for the open field test. Mice were introduced into the center of the chamber at the beginning of the test and movements were recorded and analyzed by automatic video tracking (ANY-maze 4.99) for 15 min. Locomotor activity was evaluated as the distance traveled per 5 min and the total distance.

### Three-chamber test

The apparatus consisted of a rectangular plexiglas box (60 × 40 × 20 cm) divided into three chambers. The test contained three phases: (A) Habituation (Phase 1): The left and right chambers each contained an empty wire cage (E). The test mouse was placed in the middle chamber and allowed to freely explore for 10 min. The total time spent in each chamber was measured. (B) Sociability (Phase 2): an age- and gender- matched stranger mouse (S1) was introduced to a wire cage. The test mouse was placed in the center chamber and allowed to freely explore for 10 min. The total time spent in each chamber and close interaction time with the cages were measured. Generally speaking, close interaction was defined as a 5 cm radius proximal to each wire cage. (C) Social novelty recognition (Phase 3): a new stranger mouse (S2) was introduced to the other empty cage. The test mouse was placed in the middle chamber and allowed to explore for 10 min. The total time spent in each chamber and close interaction time with the cages were measured by automatic video tracking (ANY-maze 4.99). The preference index (S1 − E) was measured as the ratio of (S1 − E) to (S1 + E) and the preference index (S2 − S1) was measured as the ratio of (S2 – S1) to (S2 + S1).

### Nesting-building test

Mice were individually housed for at least 48 h. One hour before the onset of the dark phase, a single pressed cotton square nestlet (3.0 g) was introduced in a cage. The next morning, any unused nestlet material was weighed. The quality of the nest was assessed using the following score: 1. nestlet not noticeable touched, 2. nestlet partially torn up, 3. mostly shredded but no identifiable nest site, 4. an identifiable but flat nest, and 5. a well-defined nest with walls.

### Sucrose preference test

Mice were single-housed for a week and then given two bottles of 1% sucrose for 2 days. On day 3, mice were presented with two bottles of drinking water for 2 days. On day 5, mice were exposed to two bottles filled with either 1% sucrose or water. The bottle position was switched after 24 h. Total consumption of each fluid within 48 h was measured and the sucrose preference was defined as the ratio of the consumption of sucrose solution versus the consumption of both water and sucrose solution during the 48 h test.

### Novel object recognition test

The object recognition test was performed in an open field apparatus. On the first day, the mice were introduced in the center of the open field to freely explore for 10 min. The next day, two identical objects were placed in the apparatus, then the mouse was introduced in the center of the objects and allowed to freely explore for 10 min. Thirty minutes later, one of the objects was replaced with a new object and the mice were allowed to explore for 5 min. For the modified novel object recognition test, one of the objects was replaced with a new object and the mice were allowed to explore immediately after the previous 10 min of free exploration. Exploration was considered as directing the nose at a distance <2 cm from the object and/or touching it with the nose. The exploration time spent on each of the familiar (F) object and the new (N) object was recorded manually in blind manner. Discrimination index was calculated by [(N – F)/(N + F)] × 100% for intergroup comparison.

### Temporal order memory test

Temporal order memory was performed in an open field apparatus. The mice were introduced in the center of the open field to freely explore for 10 min the day before the experiment. The temporal order memory task consisted of two sample trials and one test trial. On the first sample trial, two identical objects (later referred to as the “less recent” object) was placed in the familiar open field apparatus, then the mouse was introduced in the center of the objects and allowed to freely explore for 10 min. Thirty minutes later, the objects were replaced with different novel objects (later referred to as the “recent” object) and the mice were allowed to explore for 10 min. During the test trial, a less recent object and a recent object were placed onto positions previously encountered in the sample trials. Exploration was considered as directing the nose at a distance <2 cm from the object and/or touching it with the nose. The exploration time spent on each of the less recent (L) object and the recent (R) object was recorded manually in blind manner. Discrimination index was calculated by [(L – R)/(L + R)] × 100% for intergroup comparison.

### Delayed non-match-to-sample (DNMTS) task

Weight of mice was maintained at 80–90% of their starting weight prior to the test. Mice were exposed to the Y-maze for a period of 2–3 days and then were tested with four trials per day in the Y maze. Each trial began with a sample run, in which mice collected a food reward from a randomly chosen left or right arm while the entrance to the other arm remained closed. The trial continued after an interval of 5 s with a choice run, in which both arms were opened. The correct choice was scored when the mouse visited the arm opposite to that visited on the sample run. The percentage of correct choices averaged across a given test period for each group was calculated.

### Morris water maze

The Morris water maze assay was conducted as described in refs. ^61,62^. The Morris water maze consisted of a circular pool filled with water (21 ± 1.5 °C) and painted white. The water pool was divided into four quadrants (I, II, III, and IV). A round platform (10 cm in diameter) was submerged 1.0 cm below the water surface in the center of quadrant III (target quadrant). Spatial cues consisting of black and white posters were constantly visible from the pool. The behavior of the animal was recorded by a video camera mounted on the celling of the pool and a tracking system. The Morris water maze test involved a four-day navigation trial and a one-day probe trial. During the navigation trial, the mouse was placed into the water facing the wall of the pool from four different entry points and allowed to swim. The time taken (escape latency) to find the platform and the average swimming speed were recorded. If the mouse reached the platform and stayed for 10 s within 60 s, the test would end automatically. If the mouse could not find the platform, it would be guided to the platform by the experimenter and allowed to stay on it for 10 s, with the escape latency recorded as 60 s. One day after the last training session, the probe trial was conducted in which the platform was removed and the mice were allowed to swim freely for 60 s. The number of platform area crossings and time spent in the target quadrant (%) were recorded.

### Tail suspension test

After 30 min habituation period in the test room, mice were gently suspended by the tail for 6 min, and immobility time was measured.

### Forced swimming test

After 30 min habituation period in the test room, mice were placed in clear glass beakers filled with water for 6 min. The water temperature maintained (21 ± 1.5 °C). Immobility time was measured.

### Light-Dark box

The Light-Dark box consists of a dark chamber (~5 lux, 30 × 20 × 25 cm) and a light chamber (~600 lux, 15 × 20 × 25 cm) with a small opening allowing mice to freely move across the light and dark chambers. The mice were introduced to the center of light chamber and allowed to explore for 5 min. The time spent in the light chamber was measured.

### Elevated plus maze

The EPM apparatus consists of two open arms (30 × 5 cm) and two closed arms (30 × 5 × 20 cm) elevated 50 cm above the floor. Mice were placed in the center facing the open arms and allowed to explore for 5 min. The time spent in the open arms and entries in the open arms were measured.

### Drug administration

Risperidone (0.05 mg/kg/d, Sigma-Aldrich, USA), clozapine (1 mg/kg/d, Sigma-Aldrich, USA) and haloperidol (1 mg/kg/d, Sigma-Aldrich, USA) were dissolved in saline with acetic acid, after which the pH was adjusted to pH 6.0 with sodium hydroxide. For chronic administration, mice were treated with drugs by i.p. injection for 2 weeks. In the behavior tests, the drugs were administered 1 h before the test. For the acute administration, mice were injected with MK-801 ((+)-MK-801 hydrogen maleate, Sigma-Aldrich, USA, 0.2 mg/kg, i.p., 0.05 mg/ml in 0.9% saline), or vehicle (0.9% saline) after a 30-min habituation period in the clear plexiglas box (45 × 45 × 45 cm). Mouse behavior was automatically video-recorded for 120 min and mouse position was determined and analyzed every 5 min by automatic video tracking (ANY-maze 4.99). For chronic administration, mice were treated with MK-801 by injection (0.2 mg/kg) once daily for 2 weeks.

### Virus injection

AAV-CAG-FLEX-*Hrh1*-GFP (AAV-CAG-FLEX-GFP as control virus), AAV-EF1α-DIO-hM3Dq-mCherry, and AAV-EF1α-DIO-hM4Di-mCherry were produced by OBiO Technology (Shanghai, China). Two-month-old mice were anesthetized by intraperitoneal injection of sodium pentobarbital (50 mg/kg) and placed in a stereotaxic apparatus (RWD Life Science, Shenzhen, China). The stereotaxic coordinates were AP + 0.75 mm, ML ± 0.8 mm, and DV − 5.0 mm for HDB injection, AP − 0.5 mm, ML ± 1.8 mm, and DV − 4.4 mm for NBM injection and AP + 0.98 mm, ML ± 1.5 mm, and DV − 3.2 mm for CPu injection. A total of 600 nl of virus was injected bilaterally into HDB, NBM, and CPu within 10 min. After surgery, mice were returned to the home cage and maintained for 4 weeks to ensure virus expression before behavioral tests. To avoid the potential interference from off-target viral expression, brains from animals receiving injection were perfused, sectioned, and checked by fluorescence microscopy for GFP or mCherry expression in the targeted region. Mice that had received inaccurate viral injection were excluded from all relevant analyses. For chemogenetic modulation, CNO was administered either by i.p. injections at 1 mg/kg or by intra-mPFC injections (3 μM, 100 nL) at 30 min before the behavior test. For intra-mPFC injection, 0.30 mm diameter bilateral cannulas (RWD Life Science, Shenzhen, China) were implanted into the mPFC (AP + 2.0 mm, ML ± 0.4 mm, and DV − 2.5 mm) 1 week before the CNO delivery and behavioral tests.

### In situ hybridization by RNAscope

Mice were perfused with saline and 4% paraformaldehyde in PBS (pH 7.4). The harvested brains were fixed in 4% paraformaldehyde for another day before consecutive dehydration in 10, 20, and 30% sucrose. Brain slices with 14-μm thickness at a similar coronal position were subjected for ISH. RNAscope Multiplex Fluorescent Reagent Kit v2 (Advanced Cell Diagnostics, USA) was used for validation of the *Hrh1* knockout or duplex hybridization by combining the *Hrh1* probe C1 with a *GFP* probe C2. The patient brain samples were subjected for ISH by RNAscope Multiplex Fluorescent Reagent Kit v2 with *Hrh1* probe (Advanced Cell Diagnostics, USA). For further double labeling that combines ISH and immunofluorescence, the slices were then incubated with antibodies against ChAT (1:100, Millipore, USA), TH (1:1000, Sigma, USA), or Glutamate (1:1000, Sigma, USA). Fluorescent images were taken by 40×objective lens with Z-step using Leica SP8 laser confocal microscope. The *Hrh1* mRNA expression, ChAT expression, and the density or soma size of ChAT^+^ cholinergic neurons were quantified for neurons with nucleus by FIJI(ImageJ-win64). All data were collected and analyzed in a blind manner.

### Immunohistochemistry staining

Mice were transcardially perfused with a solution containing 0.9% NaCl at 4 °C, followed by 4% paraformaldehyde in PBS (pH 7.4). Brains were post-fixed by incubation in 4% paraformaldehyde at 4 °C overnight and cryoprotected by incubation in 30% sucrose for 48 h. Coronal sections of brain tissue 25-μm thickness were obtained using a cryostat (CryoStar NX50, Thermo Fisher). Brain slices were washed three times, 5 min each, with PBS. 41 ml antigen retrieval solution A (0.1 M sodium citrate: 29.41 g sodium citrate dehydrate with 1000 ml DDW) and 9 ml solution B (0.1 M citric acid: 21.01 g citric acid monohydrate with 1000 ml DDW) were added to 450 ml DDW to prepare the 10 mM antigen retrieval buffer. Then the 10 mM antigen retrieval buffer was transfered to a retrieval container and preheated to 90–100˚C. The slides were placed into the container with the preheated retrieval buffer. The container was put in the heat source for 8 min and then allowed to cool at room temperature for 30 min. Then sections were permeabilized with 0.1% Triton X-100 in PBS for 15 min at room temperature. After blocking by 5% normal donkey serum in PBS for 1 h at room temperature, sections were first incubated with anti-ChAT antibody (1:100, Millipore, USA), anti-GFP antibody (1:400, Abcam, USA), anti-dsRed antibody (1:200, Takara, Japan.) at 4 °C overnight and then with AlexaFluor-conjugated secondary antibody (1:400, Jackson ImmunoResearch Laboratories, USA) at room temperature. Fluoroshield^TM^ with DAPI (Sigma-Aldrich, USA) was used as a nuclear stain. All sections at a similar coronal position are observed under a Leica SP8 laser confocal microscope. The images were measured by FIJI(ImageJ-win64) and all data were collected and analyzed in a blind manner.

### Western blot

The brain tissues were homogenized in RIPA buffer (20 mmol/L TRIS-HCl pH = 7.5, 150 mmol/L NaCl, 1 mmol/L EDTA, 1% Triton X-100, 0.5% sodium deoxycholate, 0.1% SDS, 20 mmol/L NaF, and 1 mmol/L PMSF). Supernatants were collected after centrifugation for 10 min at 13,000×*g*. An aliquot of 40 µg total protein from each sample was run on 10% SDS/PAGE gels and transferred to a nitrocellulose membrane, which was then blocked with 5% nonfat milk in PBS (pH 7.4). The membranes were incubated with primary antibodies against ChAT (1:1000, AB144P, Millipore) or GAPDH (1:3000, Kangchen, China) at 4 °C overnight. Secondary antibodies conjugated with horseradish peroxidase against either goat or mouse IgG (1:3000, Multi Sciences, China) were performed for 2 h at room temperature and blots were exposed to ECL Western blotting detection reagents (Multi Sciences, China). Signals were acquired with a gel documentation system (Tanon 5200, China) and quantified with Image Pro Plus (version 6.0).

### Microdialysis and HPLC-MS/MS

For extracelluar ACh in PFC, microdialysis probe (MAB, Sweden) was inserted into the mPFC (AP + 2.0 mm, ML + 0.4 mm, and DV − 2.5 mm) through the guide cannula 1 week before the experiment. Microdialysis was started by connecting the probe inlet to a microinjection pump system (CMA, Sweden) that circulated the probe continuously with artificial CSF (125 mM NaCl, 2.5 mM KCl, 1.26 mM CaCl_2_, 1.18 mM MgCl_2_, and 10 μM neostigmine to prevent ACh degradation) at a rate of 1 μl/min. The perfusion exudate from the first 60 min was discarded and then samples were collected every 1 h thereafter. A total of four samples were collected on each sampling day. All of the microdialysis samples were immediately frozen at −80 °C and stored until they were analyzed by HPLC-MS/MS. For testing the ACh content in tissues, the BF and CPu were homogenized in five volumes (g/ml) acetonitrile on ice and the samples were centrifuged (20,000×*g*, 4 °C) for 20 min. The supernatants were diluted four times with ddH_2_O and repeatedly centrifuged (20,000×*g*, 4 °C) for 20 min. The supernatants were stored at −80 °C until analysis.

The HPLC system consisted of chromatographic column (Inertsil ODS-EP, 150 × 4.6 mm, 5 μm) maintained at 30 °C. Chromatographic separation was achieved with gradient elution using a complex gradient, mobile phase A (0.05% formic acid in water), mobile phase B (0.05% formic acid in methanol). The HPLC gradient program was as follows: 45% B → 80% B at 0.10–2.00 min; 80% B → 100% B at 2.00–4.00 min; 100% B → 45% B at 4.00–4.01 min; 45% B at 4.01–5.00 min. The flow rate was 0.5 mL/min and the injection volume was 5 μl. Mass spectrometric detection was in the multiple reaction monitoring modes by using an electrospray positive ionization.

### Electrophysiology

The brain was quickly removed and immersed in ice-cold artificial cerebrospinal fluid (ACSF) containing in mM: 120 NaCl, 11 Dextrose, 2.5 KCl, 1.28 MgSO_4_, 3.3 CaCl_2_, 1 NaH_2_PO_4_, and 14.3 NaHCO_3_, which was constantly bubbled with 95% O_2_ and 5% CO_2_. Coronal slices at 300 µm thickness were cut using a vibratome (VT1000 mol/L/E, Leica) and incubated at 25 °C for 1 h. The slices were transferred into a recording chamber at 25 °C for patch clamp recording. The patch pipette (5 to 10 mol/LΩ resistance) was filled with recording solution (containing in mM: 140 K-gluconate, 5 NaCl, 0.2 EGTA, 2 Mg-ATP, 10 HEPES, and 0.2% biocytin). Signals were amplified and recorded by an HEKA EPC10 amplifier (HEKA Instruments, Germany). To test the action potential threshold of neurons being recorded, episodic currents were injected under the current clamp configuration in 5 pA increments from 0 pA to depolarizing 100 pA.

To record spontaneous synaptic currents, a low divalent ion ACSF (in mM: 125 NaCl, 3.5 KCl, 1.25 NaH_2_PO_4_, 0.5 MgCl_2_, 26 NaHCO_3_, 25 Dextrose, and 1 CaCl_2_) was used. Using pipettes filled with cesium-based internal fluid (in mM: 100 CsCH_3_SO_3_, 20 KCl, 10 HEPES, 4 Mg-ATP, 0.3 Tris-GTP, 7 Tris2-Phosphocreatine, and 3 QX-314), sEPSCs were recorded at a holding potential of −60 mV and sIPSCs were recorded at a holding potential of +10 mV. Individual events were counted and analyzed with MiniAnalysis software (version 6.0.3).

### Statistical analysis

Number of experimental replicates (*n*) is indicated in figure legend and refers to the number of experimental subjects independently treated in each experimental condition. No statistical methods were used to pre-determine sample size, or to randomize. All datasets were tested for Gaussian distribution using a Shapiro–Wilk normality test. Two datasets were statistically compared using a Student’s *t* test if the null hypothesis of normal distribution was not rejected. ANOVA tests were used when comparing more than two normally distributed datasets. In case of non-normal data distribution, non-parametric tests were used: Mann–Whitney *U* test was used for single comparisons, the Kruskal–Wallis test for one-way analysis of variance, and the Scheirer Ray Hare test for two-way analysis of variance. Statistical analyses were carried out using Prism (version 7.0) or SPSS (version 17.0). A statistical significance threshold was set at 0.05, and significance levels are presented as *P ≤ 0.05, **P ≤ 0.01, or ***P ≤ 0.001 in all figures.

### Reporting Summary

Further information on research design is available in the Nature Research Reporting Summary linked to this article.

## Supplementary information

Description of Additional Supplementary Files

Supplementary Information

Supplementary Data 1

Supplementary Data 2

Supplementary Data 3

Reporting Summary

## Data Availability

The datasets generated and/or analyzed during the current study are available from the corresponding author upon reasonable request. Source data are provided with this paper.

## Source data

Source Data
