# Supplementary material for: Histamine H1 receptor deletion in cholinergic neurons induces sensorimotor gating ability deficit and social impairments in mice
Source: Nat Commun. 2021 Feb 18;12:1142. doi: 10.1038/s41467-021-21476-x (PMC7893046; doi:10.1038/s41467-021-21476-x)
Supplement: Supplementary file 1 — Description of Additional Supplementary Files [file 41467_2021_21476_MOESM1_ESM.docx]

**Description of Additional Supplementary Files**

File Name: Supplementary Data 1

Description: Clinico-pathological information of NBB patients with schizophrenia and control subjects

File Name: Supplementary Data 2

Description: Statistical Data

File Name: Supplementary Data 3

Description: The PCR primers sequences designed for the Hrh1 gen
