# Supplementary material for: Histamine H1 receptor deletion in cholinergic neurons induces sensorimotor gating ability deficit and social impairments in mice
Source: Nat Commun. 2021 Feb 18;12:1142. doi: 10.1038/s41467-021-21476-x (PMC7893046; doi:10.1038/s41467-021-21476-x)
Supplement: Supplementary file 2 — Supplementary Information [file 41467_2021_21476_MOESM2_ESM.pdf]

Histamine H_1 receptor deletion in cholinergic neurons induces sensorimotor gating ability deficit and social impairments in mice

Cheng et al.

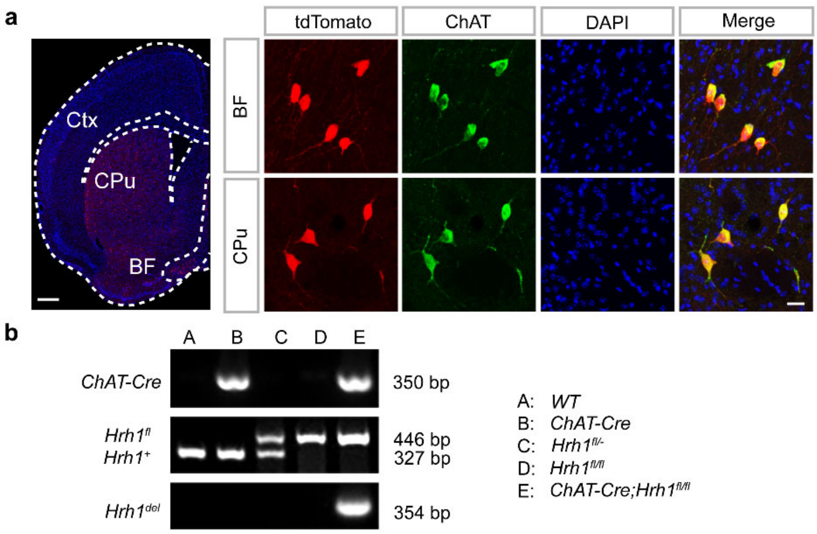

Supplementary Fig 1. The distribution of Cre/tdTomato in *ChAT-Cre;Ai14* mice and genotyping of *ChAT-Cre;Hrh1*$^{fl/fl}$ mice. (a) Representative micrographs of Cre/tdTomato distribution (red) in the BF and CPu. Slices were obtained from *ChAT-Cre;Ai14* mice and stained with antibody against ChAT (green). Left: scale bar, 500 μm. Right: scale bar, 20 μm. **(b)** Genotyping of *ChAT-Cre;Hrh1*$^{fl/fl}$ mice and control mice. The pair of Hrh1-loxptF and Hrh1-loxptR primers generates a 327-base pair (bp) product for the wild-type allele or a 446 bp product for the loxP-flanked allele for *Hrh1*. The pair of Hrh1-loxptF and Hrh1-FRT-tR primers generates a 354 bp product for the deleted allele for *Hrh1*. The *ChAT-Cre* primers generate a 350 bp band.

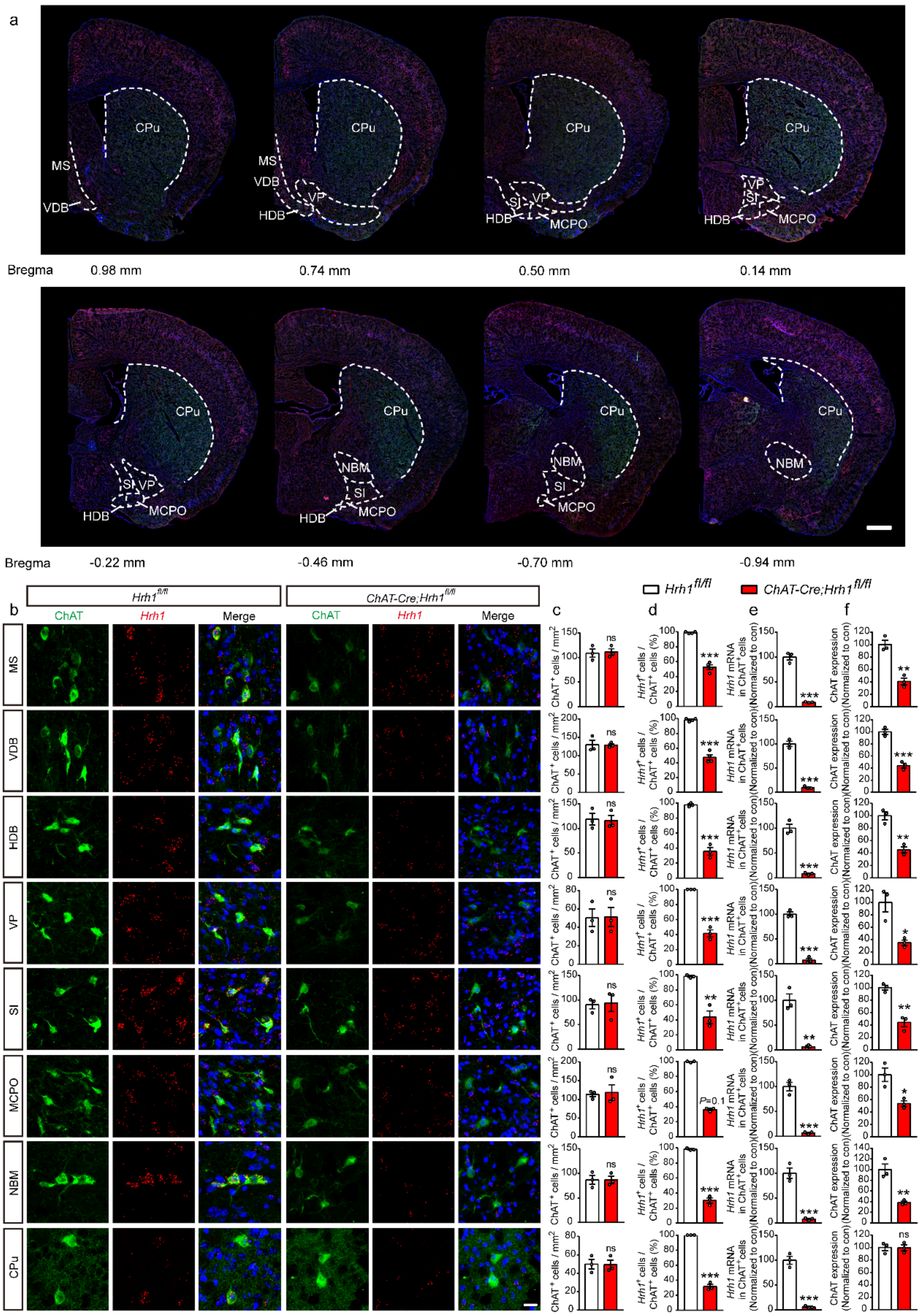

Supplementary Fig 2. Stereological estimates of regional quantification of *Hrh1* mRNA, ChAT expression and cell density of cholinergic cells in the BF of *Hrh1*$^{fl/fl}$ and *ChAT-Cre;Hrh1*$^{fl/fl}$ mice. (a) Representative photomicrographs of the analyzed areas in parasagittal sections of substantial rostral-caudal part of the BF. Scale bar, 500 μm. **(b)** *In situ* hybridization of H_1R mRNA together with immunostaining of ChAT and visualization of nuclei by DAPI in BF and CPu to confirm the deletion of H_1R in cholinergic neurons, ChAT expression and cell density

of ChAT⁺ cells. Scale bar, 20 μm. **(c)** Quantitative analysis of cell density in the BF and CPu of *ChAT-Cre;Hrh1*$^{fl/fl}$ and *Hrh1*$^{fl/fl}$ mice. **(d)** Percentage of *Hrh1*$^{+}$ cells in ChAT^{+} cells in the BF and CPu of *ChAT-Cre;Hrh1*$^{fl/fl}$ and *Hrh1*$^{fl/fl}$ mice. **(e)** Quantitative analysis of *Hrh1* mRNA expression in ChAT^{+} cells in the BF and CPu of *ChAT-Cre;Hrh1*$^{fl/fl}$ and *Hrh1*$^{fl/fl}$ mice. **(f)** Quantitative analysis of ChAT expression in the BF and CPu of *ChAT-Cre;Hrh1*$^{fl/fl}$ and *Hrh1*$^{fl/fl}$ mice. All data are presented as mean ± s.e.m. and error bars represent s.e.m. *$P \leq 0.05$, **$P \leq 0.01$, ***$P \leq 0.001$, ns, nonsignificant. See also Supplementary Data 2 for further statistical information. Source data are provided as a Source Data file.

con: *Hrh1*$^{fl/fl}$ mice. MS: medial septal nucleus; VDB: nucleus of the vertical limb of the diagonal band; HDB: nucleus of the horizontal limb of the diagonal band; VP: ventral pallidum; SI: substantia innominata; MCPO: magnocellular preoptic nucleus; NBM: nucleus basalis magnocellularis; CPu: caudate putamen (striatum).

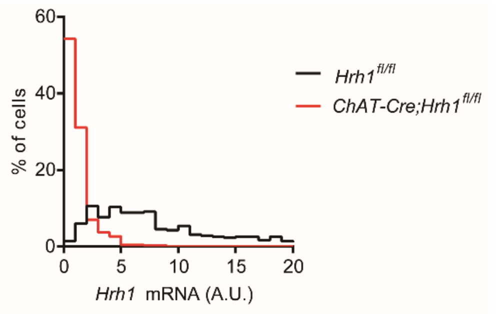

Supplementary Fig 3. Evaluation of *Hrh1* mRNA expression in ChAT^{+} cells. Histograms showing *Hrh1* mRNA expression distribution in ChAT^{+} cells of *Hrh1*$^{fl/fl}$ and *ChAT-Cre;Hrh1*$^{fl/fl}$ mice. A.U., Arbitrary Unit. See also Supplementary Data 2 for further statistical information. Source data are provided as a Source Data file.

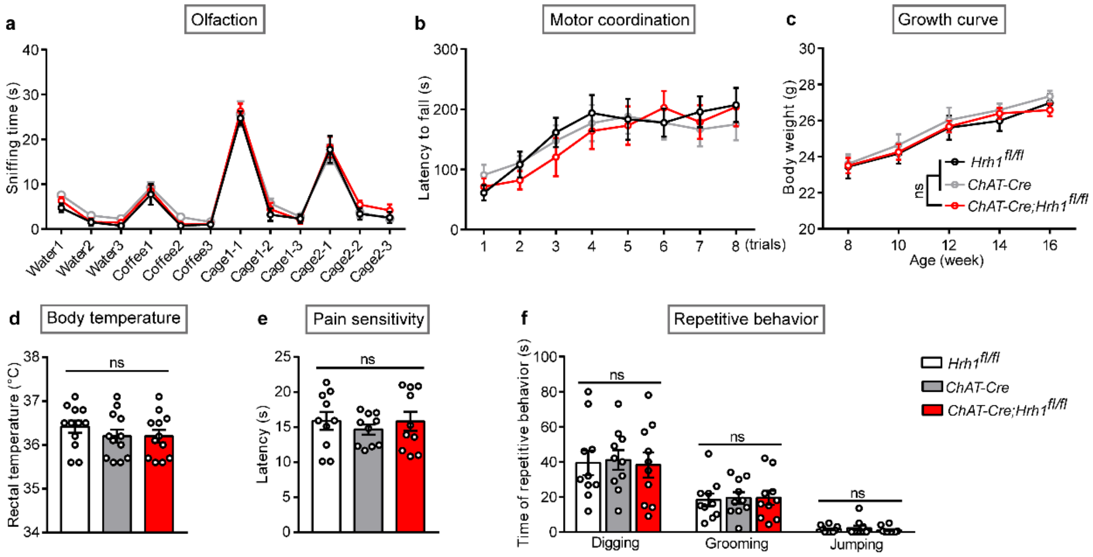

Supplementary Fig 4. Deletion of histamine H_1R in cholinergic neurons does not show alteration in physiological functions in mice. (a-f) Olfactory function, motor coordination function, growth curve, body temperature, pain sensitivity, and repetitive behavior in *ChAT-Cre;Hrh1*$^{fl/fl}$ mice, control *Hrh1*$^{fl/fl}$ and *ChAT-Cre* mice. **(a)** Ability to recognize social and non-social odors, as assessed by the olfactory habituation/dishabituation test. **(b)** Motor coordination function tested by accelerated rotarod. **(c)** Growth curve from 8 to 16 weeks of age. **(d)** The body temperature was tested by a rectal thermometer. **(e)** Pain sensitivity was assessed by the hot plate test. **(f)** Time spent in repetitive behaviors including digging, grooming and jumping. All data are presented as mean ± s.e.m. and error bars represent s.e.m. ns, nonsignificant. See also Supplementary Data 2 for further statistical information. Source data are provided as a Source Data file.

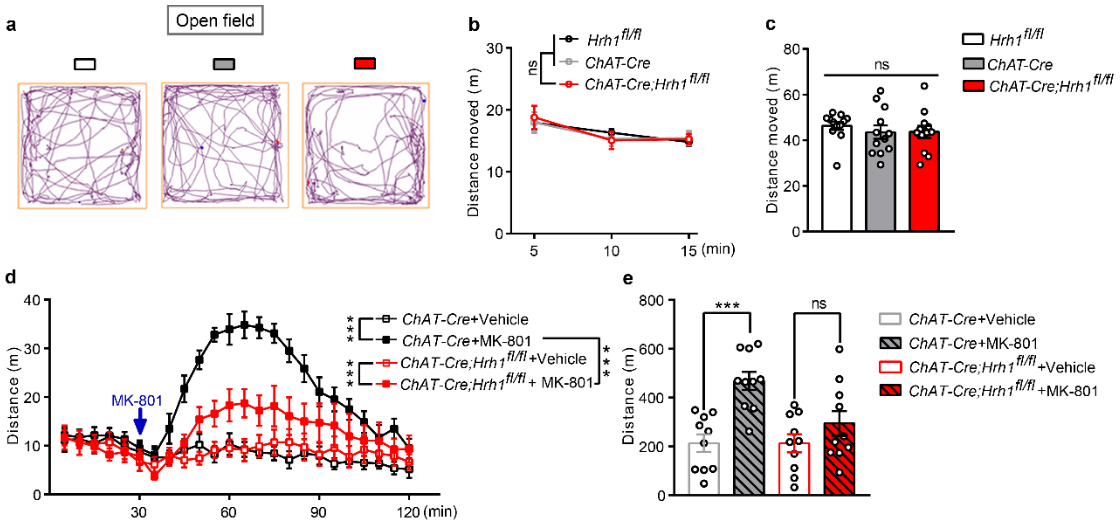

Supplementary Fig 5. The locomotor activity is normal, while the MK-801 induced hyperlocomotion is attenuated in *ChAT-Cre;Hrh1$^{fl/fl}$* mice. (a) Representative trajectories of *ChAT-Cre;Hrh1$^{fl/fl}$* mice, control *Hrh1$^{fl/fl}$* and *ChAT-Cre* mice. **(b)** Locomotor activity assessed by the distance traveled in the open field for every 5 min. **(c)** The cumulative distance traveled in the open field for 15 min. **(d)** The distance traveled in the open field for every 5 min was quantified and MK-801 was administrated 30 min after free exploration. **(e)** The cumulative distance for 120 min in (d). All data are presented as mean ± s.e.m. and error bars represent s.e.m. ***$P \leq 0.001$, ns, nonsignificant. See also Supplementary Data 2 for further statistical information. Source data are provided as a Source Data file.

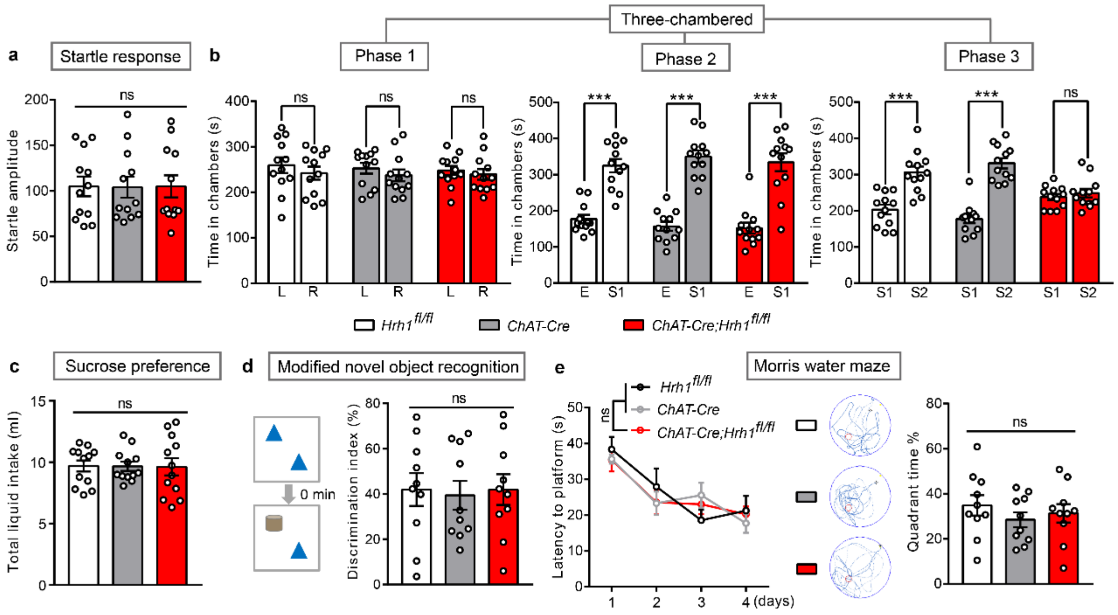

Supplementary Fig 6. Behavioral characteristics in *ChAT-Cre;Hrh1*$^{fl/fl}$ mice. (a) Startle response was tested as the amplitude at the 120 dB startle pulse. **(b)** Time in chambers during phase 1, 2 and 3 in the three-chamber test. L: left; R: right; E: empty wire cage; S1: a gender-matched stranger mouse; S2: a new gender-matched stranger mouse. **(c)** The basal total liquid intake for 48 h. **(d)** The instant recognition ability was tested in the modified novel object recognition test without a gap between free exploration and a subsequent test. **(e)** Spatial working memory was tested by the Morris water maze. The latency in time to find the platform during training period, time spent in quadrant with platform during test period, and representative trajectories in the middle panel. All data are presented as mean ± s.e.m. and error bars represent s.e.m. ****P*≤0.001, ns, nonsignificant. See also Supplementary Data 2 for further statistical information. Source data are provided as a Source Data file.

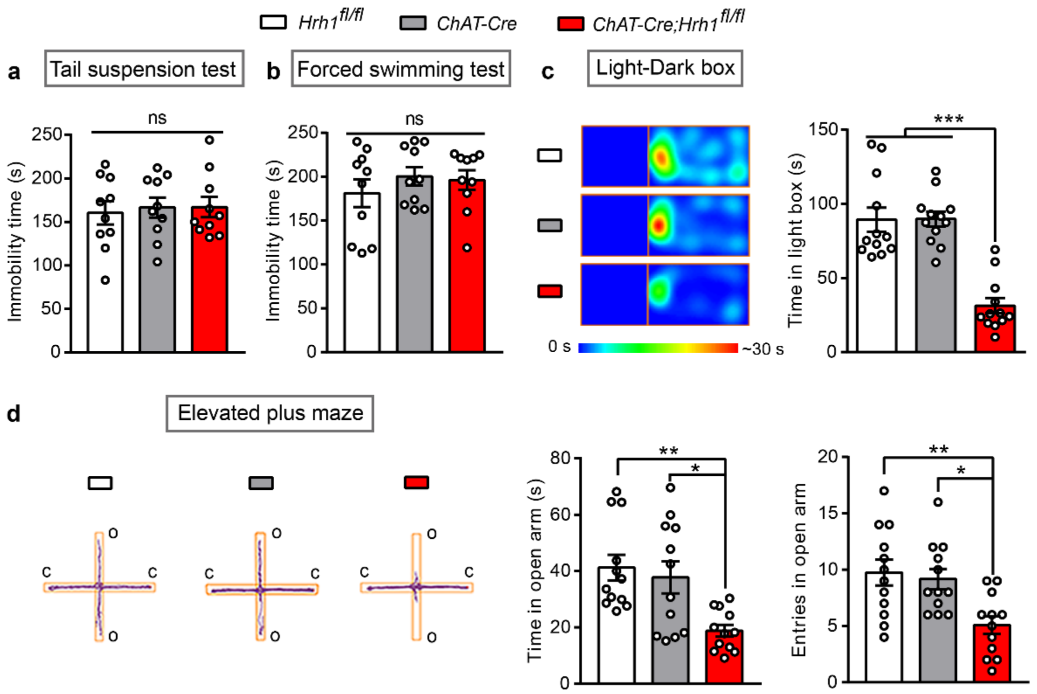

Supplementary Fig 7. Deletion of histamine H_1 receptors in cholinergic neurons induces anxiety-like but not depression-like behaviors in mice. (a) Immobility time in the tail suspension test. **(b)** Immobility time in the forced swimming test. **(c)** Representative heat map images and time in the light box during the light-dark box test. **(d)** Examples of the performance of control and *ChAT-Cre;Hrh1$^{fl/fl}$* mice in the EPM test in the left panel. c, closed arm; o, open arm. The time in the open arm during EPM test. The entries in the open arm during EPM test. All data are presented as mean ± s.e.m. and error bars represent s.e.m. **P*≤0.05, ***P*≤ 0.01, ****P*≤0.001, ns, nonsignificant. See also Supplementary Data 2 for further statistical information. Source data are provided as a Source Data file.

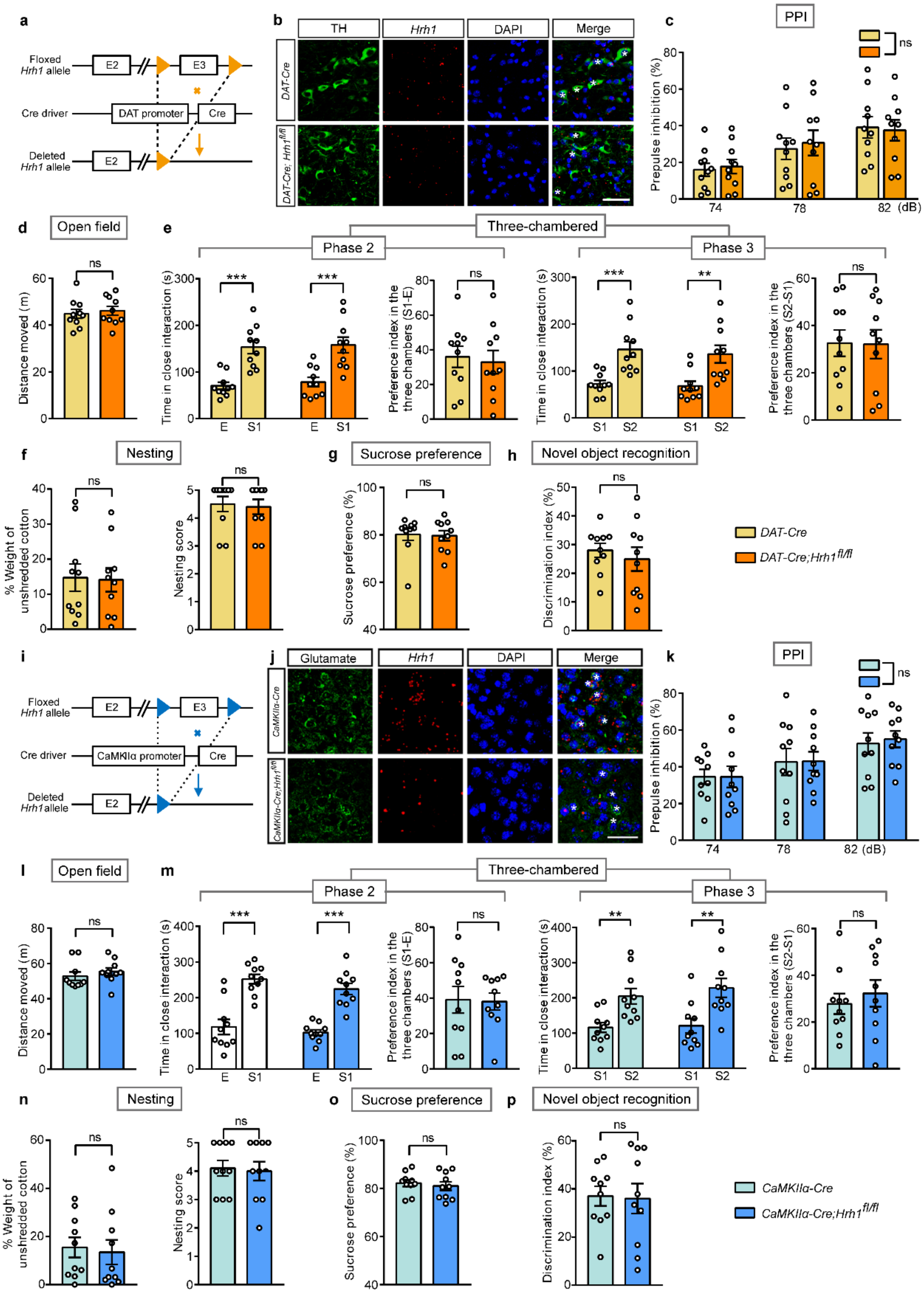

Supplementary Fig 8. Deletion of histamine H_1R in dopaminergic or glutamatergic neurons does not induce sensorimotor gating ability deficit, hyperlocomotion, social impairments, anhedonia or cognitive impairments in mice. (a) Schematic diagram of the generation of *DAT-Cre;Hrh1*$^{fl/fl}$ mice. **(b)** RNAscope *in situ* hybridization of *Hrh1* mRNA together with immunostaining of tyrosine hydroxylase (TH) and visualization of nuclei by DAPI in ventral tegmental area (VTA) to confirm the deletion of H_1R in dopaminergic neurons. Scale bar, 40 µm. **(c-h)** Sensorimotor gating, locomotion, social behavior, hedonic function and cognitive behavior

in control *DAT-Cre* mice and *DAT-Cre;Hrh1$^{fl/fl}$* mice. **(c)** Percentage of prepulse inhibition of the auditory startle reflex across different prepulse intensities. **(d)** Locomotor activity assessed by the total distance traveled in open field within 15 min. **(e)** Time in close interaction and preference index during sociability testing (phase 2), when exposed to a stranger mouse S1. Time in close interaction and preference index during subsequent social novelty recognition testing (phase 3), when exposed to a new stranger mouse S2 together with S1. **(f)** The percentage of the weight of unshredded cotton and nesting score in nest-building test. **(g)** Test for sucrose preference as a percentage of all fluid intake within a 48 h period. **(h)** Discrimination index in novel object recognition test. **(i)** Schematic diagram of the generation of *CaMKIIα-Cre;Hrh1$^{fl/fl}$* mice. **(j)** RNAscope *in situ* hybridization of *Hrh1* mRNA together with immunostaining of glutamate and visualization of nuclei by DAPI in sensory cortex and motor cortex to confirm the deletion of H_1R in glutamatergic neurons. Scale bar, 30 µm. **(k-p)** Sensorimotor gating, locomotion, social behavior, hedonic function and cognitive behavior in control *CaMKIIα-Cre* mice and *CaMKIIα-Cre;Hrh1$^{fl/fl}$* mice. **(k)** Percentage of prepulse inhibition of the auditory startle reflex across different prepulse intensities. **(l)** Locomotor activity assessed by the total distance traveled in open field within 15 min. **(m)** Time in close interaction and preference index during sociability testing (phase 2), when exposed to a stranger mouse S1. Time in close interaction and preference index during subsequent social novelty recognition testing (phase 3), when exposed to a new stranger mouse S2 together with S1. **(n)** The percentage of the weight of unshredded cotton and nesting score in nest-building test. **(o)** Test for sucrose preference as a percentage of all fluid intake within a 48 h period. **(p)** Discrimination index in novel object recognition test. All data are presented as mean ± s.e.m. and error bars represent s.e.m. $^{**}P \leq 0.01$, $^{***}P \leq 0.001$, ns, nonsignificant. See also Supplementary Data 2 for further statistical information. Source data are provided as a Source Data file.

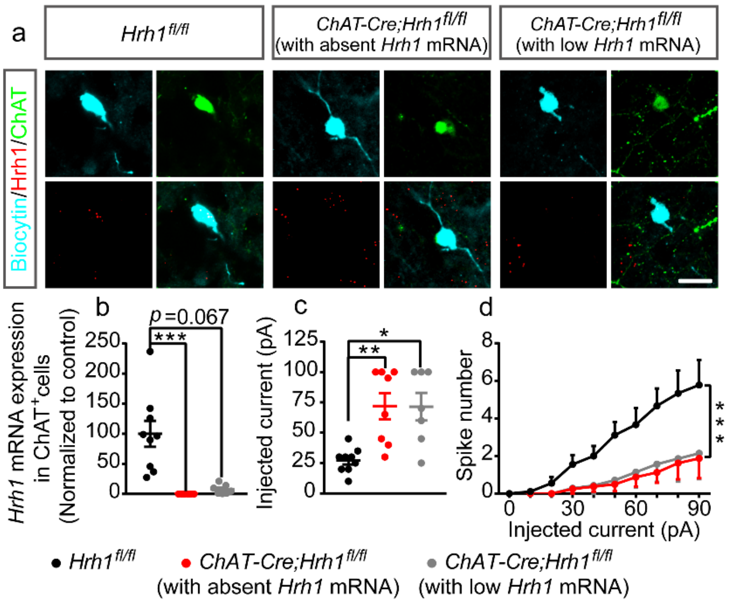

Supplementary Fig 9. Assessment of the relationship between *Hrh1* mRNA expression and excitability in ChAT^{+} cells by *in situ* hybridization and immunohistochemistry following patch clamp recordings. (a) Representative images of cholinergic neurons filled with biocytin during recording in the HDB of *Hrh1*$^{fl/fl}$ and *ChAT-Cre;Hrh1*$^{fl/fl}$ mice. Scale bar, 20 µm. **(b)** Quantitative analysis of *Hrh1* mRNA expression in ChAT^{+} cells. **(c)** Threshold current to elicit action potential with the increase of injected currents in HDB cholinergic neurons recorded by whole-cell patch-clamp. **(d)** Spike numbers with the increase of injected currents in HDB cholinergic neurons recorded by whole-cell patch-clamp. All data are presented as mean ± s.e.m. and error bars represent s.e.m. **P*≤0.05, ***P*≤ 0.01, ****P*≤0.001, ns, nonsignificant. See also Supplementary Data 2 for further statistical information. Source data are provided as a Source Data file.

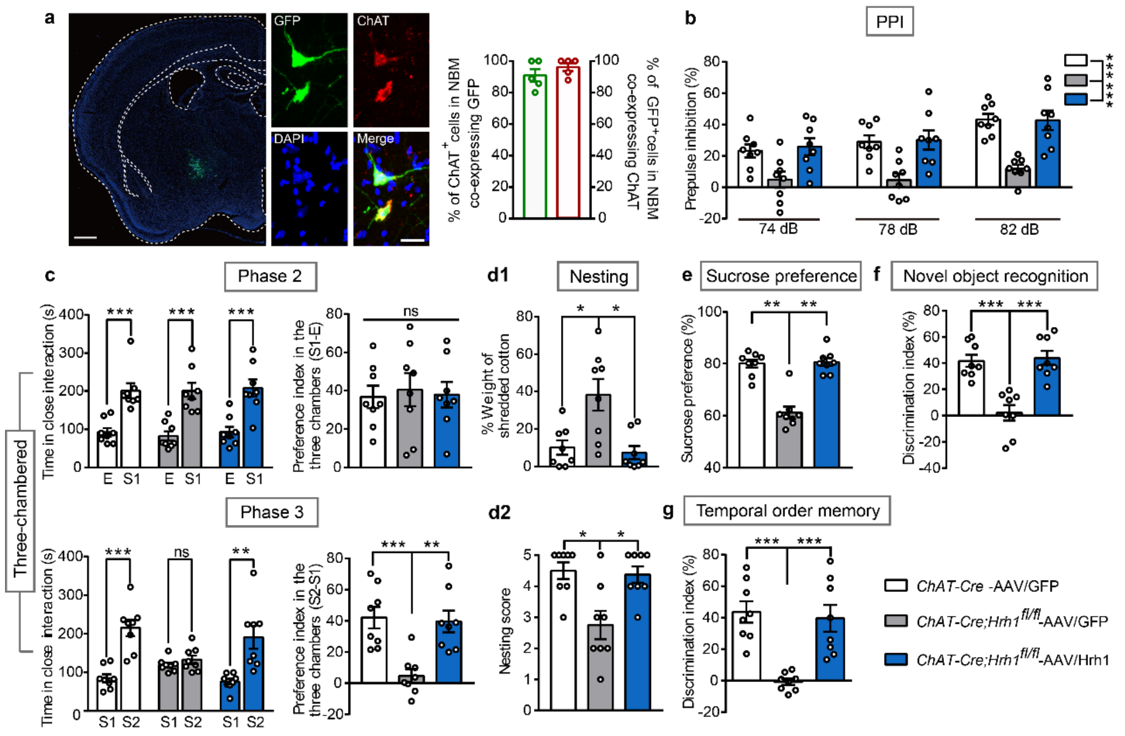

Supplementary Fig 10. Re-expression of histamine H_1R in cholinergic neurons in the NBM of BF rescues behavioral deficits manifested in *ChAT-Cre;Hrh1$^{fl/fl}$* mice. (a) Representative images of H_1R-GFP expression in the NBM of BF in *ChAT-Cre;Hrh1$^{fl/fl}$* mice after the microinjection of AAV-CAG-FLEX-*Hrh1*-GFP (AAV/Hrh1), enlarged images of GFP and immunostaining of ChAT. The percentage of ChAT$^+$ cholinergic neurons co-expressing GFP and percentage of GFP$^+$ cells co-expressing ChAT in the NBM were quantified. Left: scale bar, 500 µm. Right: scale bar, 20 µm. n=5 mice. **(b-g)** Sensorimotor gating, social behavior, hedonic function, and cognitive behavior in control *ChAT-Cre* mice injected with AAV-CAG-FLEX-GFP (AAV/GFP), and *ChAT-Cre;Hrh1$^{fl/fl}$* mice either injected with AAV/GFP or AAV/Hrh1. **(b)** Percentage of prepulse inhibition of the auditory startle reflex across different prepulse intensities. **(c)** Time in close interaction and preference index during sociability testing (phase 2) when exposed to a stranger mouse S1. Time in close interaction and preference index during subsequent social novelty recognition testing (phase 3) when exposed to a new stranger mouse S2 together with S1. **(d)** The percentage of the weight of cotton left unshredded and nesting score in nest-building test. **(e)** Test for sucrose preference as a percentage of all fluid intake within a 48 h period. **(f)** Discrimination index in novel object recognition test. **(g)** Discrimination index in temporal order memory test. All data are presented as mean ± s.e.m. and error bars represent s.e.m. *P≤0.05, **P≤ 0.01, ***P≤0.001, ns, nonsignificant. See also Supplementary Data 2 for further statistical information. Source data are provided as a Source Data file.

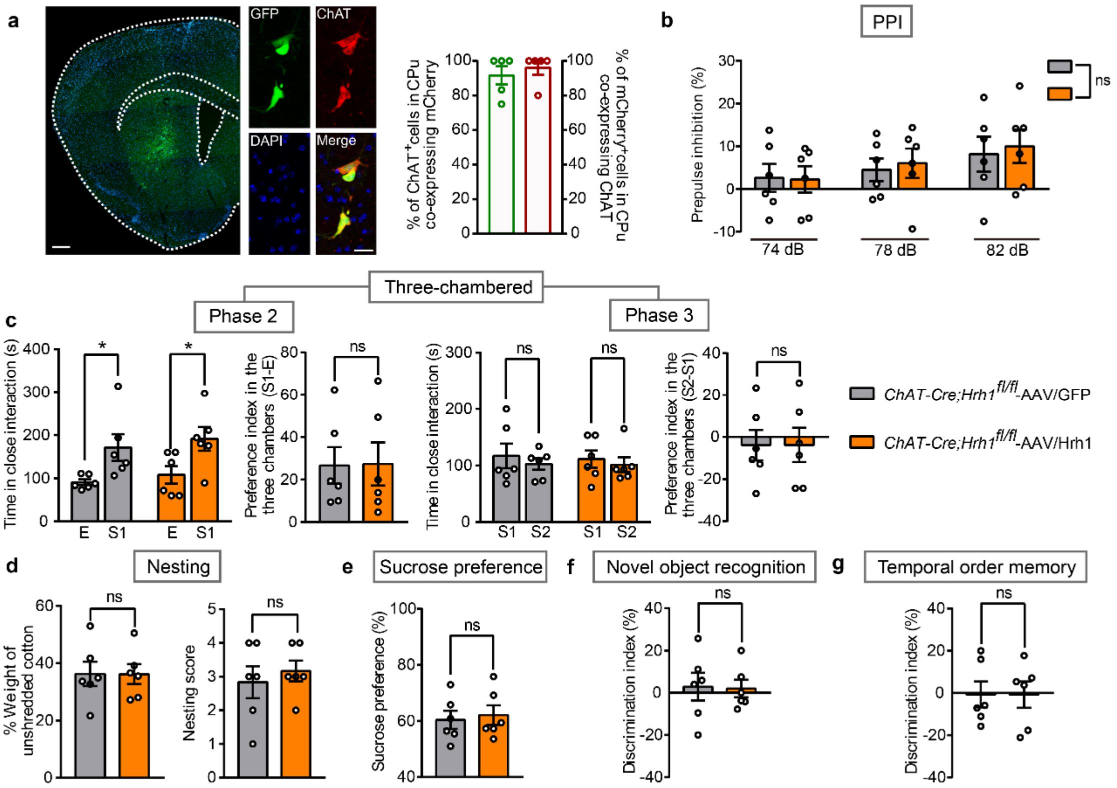

Supplementary Fig 11. Re-expression of histamine H_1R in CPu cholinergic neurons has no effect on behavioral deficits manifested in *ChAT-Cre;Hrh1$^{fl/fl}$* mice. (a) Representative images of H_1R-GFP expression in the CPu of *ChAT-Cre;Hrh1$^{fl/fl}$* mice after the microinjection of AAV-CAG-FLEX-*Hrh1*-GFP (AAV/Hrh1), enlarged images of GFP and immunostaining of ChAT. The percentage of $ChAT^+$ cholinergic neurons co-expressing GFP and percentage of GFP^+ cells co-expressing ChAT in CPu were quantified. Left: scale bar, 500 μm. Right: scale bar, 20 μm. n=5 mice. **(b-g)** Sensorimotor gating, social behavior, hedonic function and cognitive behavior in *ChAT-Cre;Hrh1$^{fl/fl}$* mice either injected with AAV-CAG-FLEX-GFP (AAV/GFP) or AAV/Hrh1. **(b)** Percentage of prepulse inhibition of the auditory startle reflex across different prepulse intensities. **(c)** Time in close interaction and preference index during sociability testing (phase 2), when exposed to a stranger mouse S1. Time in close interaction and preference index during subsequent social novelty recognition testing (phase 3), when exposed to a new stranger mouse S2 together with S1. **(d)** The percentage of the weight of unshredded cotton and nesting score in nest-building test. **(e)** Test for sucrose preference as a percentage of all fluid intake within a 48 h period. **(f)** Discrimination index in novel object recognition test. **(g)** Discrimination index in temporal order memory test. All data are presented as mean ± s.e.m. and error bars represent s.e.m. *P≤0.05, ns, nonsignificant. See also Supplementary Data 2 for further statistical information. Source data are provided as a Source Data file.

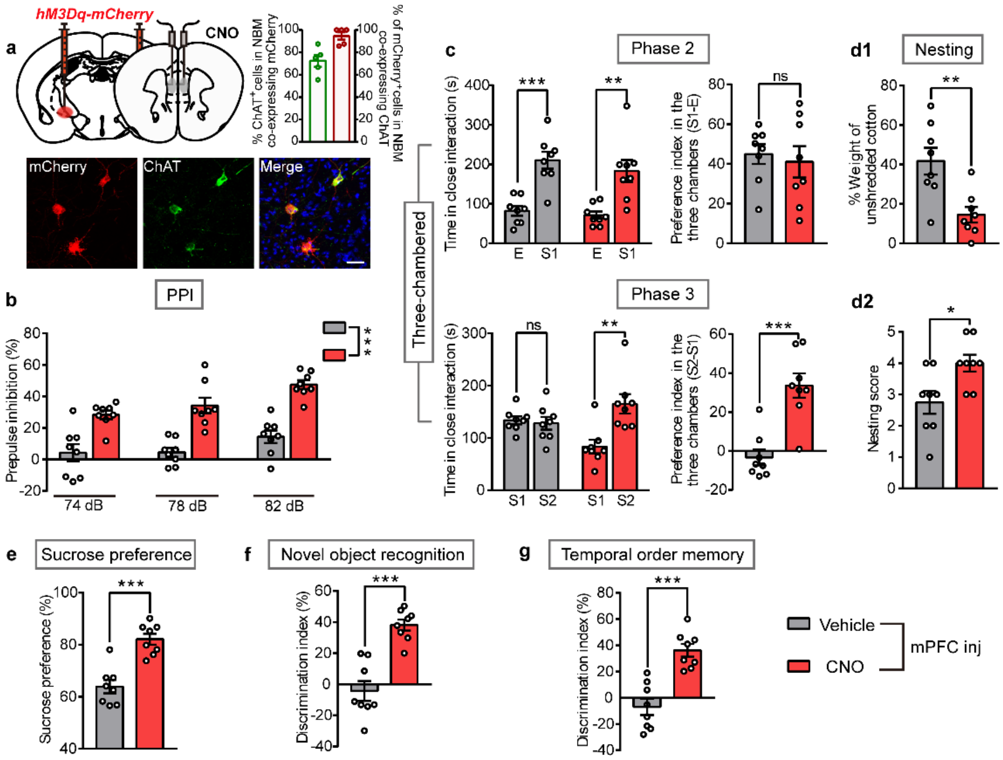

Supplementary Fig 12. Chemogenetic activation of cholinergic neurons in the NBM of BF rescues behavioral deficits in *ChAT-Cre;Hrh1$^{fl/fl}$* mice. (a) Schematic diagram of microinjection of AAV-EF1α-DIO-hM3Dq-mCherry (hM3D-mCherry) in the NBM of BF in *ChAT-Cre;Hrh1$^{fl/fl}$* mice, and representative images of hM3Dq-mCherry expression in NBM ChAT^{+} cholinergic neurons. The percentage of ChAT^{+} cholinergic cells co-expressing mCherry and percentage of mCherry expressed cells co-labeling ChAT were quantified. Scale bar, 30 μm. n=5 mice. **(b-g)** Sensorimotor gating, social behavior, hedonic function and cognitive behavior in *ChAT-Cre;Hrh1$^{fl/fl}$* mice injected with hM3D-mCherry. **(b)** Percentage of prepulse inhibition of the auditory startle reflex across different prepulse intensities. **(c)** Time in close interaction and preference index during sociability testing (phase2), when exposed to a stranger mouse S1. Time in close interaction and preference index during subsequent social novelty recognition testing (phase 3), when exposed to a new stranger mouse S2 together with S1. **(d)** The percentage of the weight of unshredded cotton and nesting score in nest-building test. **(e)** Test for sucrose preference as a percentage of all fluid intake within a 48 h period. **(f)** Discrimination index in novel object recognition test. **(g)** Discrimination index in temporal order memory test. All data are presented as mean ± s.e.m. and error bars represent s.e.m. *P≤0.05, **P≤ 0.01, ***P≤0.001, ns, nonsignificant. See also Supplementary Data 2 for further statistical information. Source data are provided as a Source Data file.
