# Supplementary material for: Histamine H1 receptor deletion in cholinergic neurons induces sensorimotor gating ability deficit and social impairments in mice
Source: Nat Commun. 2021 Feb 18;12:1142. doi: 10.1038/s41467-021-21476-x (PMC7893046; doi:10.1038/s41467-021-21476-x)
Supplement: Supplementary file 6 — Reporting Summary [file 41467_2021_21476_MOESM6_ESM.pdf]

nature research

Corresponding author(s): Zhong Chen, Weiwei Hu

Last updated by author(s): Jan 16, 2021

Reporting Summary

Nature Research wishes to improve the reproducibility of the work that we publish. This form provides structure for consistency and transparency in reporting. For further information on Nature Research policies, see our Editorial Policies and the Editorial Policy Checklist.

Statistics

For all statistical analyses, confirm that the following items are present in the figure legend, table legend, main text, or Methods section.

n/a	Confirmed	
☐	☒	The exact sample size (n) for each experimental group/condition, given as a discrete number and unit of measurement
☐	☒	A statement on whether measurements were taken from distinct samples or whether the same sample was measured repeatedly
☐	☒	The statistical test(s) used AND whether they are one- or two-sided<br>*Only common tests should be described solely by name; describe more complex techniques in the Methods section.*
☐	☒	A description of all covariates tested
☐	☒	A description of any assumptions or corrections, such as tests of normality and adjustment for multiple comparisons
☐	☒	A full description of the statistical parameters including central tendency (e.g. means) or other basic estimates (e.g. regression coefficient) AND variation (e.g. standard deviation) or associated estimates of uncertainty (e.g. confidence intervals)
☐	☒	For null hypothesis testing, the test statistic (e.g. F, t, r) with confidence intervals, effect sizes, degrees of freedom and P value noted<br>*Give P values as exact values whenever suitable.*
☒	☐	For Bayesian analysis, information on the choice of priors and Markov chain Monte Carlo settings
☐	☒	For hierarchical and complex designs, identification of the appropriate level for tests and full reporting of outcomes
☒	☐	Estimates of effect sizes (e.g. Cohen's d, Pearson's r), indicating how they were calculated

Our web collection on statistics for biologists contains articles on many of the points above.

Software and code

Policy information about availability of computer code

Data collection	Behavioral assays: Data were collected blindly using the behavior tracking software ANY-maze (version 4.99). Confocal images were captured with Leica SP8. In vitro electrophysiology was performed by using an EPC10 patch-clamp amplifier (HEKA Instruments; Germany).
Data analysis	Image analyses and quantification were performed using FIJI(Image J-win64). Individual events were counted and analyzed with MiniAnalysis software (version 6.0.3). Signals of Western blot were quantified with Image Pro Plus(version 6.0). Statistical comparisons were performed using Prism (version 7.0) or SPSS (version 17.0) with appropriate methods as indicated in figure legends.

For manuscripts utilizing custom algorithms or software that are central to the research but not yet described in published literature, software must be made available to editors and reviewers. We strongly encourage code deposition in a community repository (e.g. GitHub). See the Nature Research guidelines for submitting code & software for further information.

Data

Policy information about availability of data

All manuscripts must include a data availability statement. This statement should provide the following information, where applicable:

- Accession codes, unique identifiers, or web links for publicly available datasets
- A list of figures that have associated raw data
- A description of any restrictions on data availability

The datasets generated and/or analysed during the current study are available from the corresponding author upon reasonable request. Source data are provided with this paper.

Field-specific reporting

Please select the one below that is the best fit for your research. If you are not sure, read the appropriate sections before making your selection.

- [x] Life sciences
- [ ] Behavioural & social sciences
- [ ] Ecological, evolutionary & environmental sciences

For a reference copy of the document with all sections, see nature.com/documents/nr-reporting-summary-flat.pdf

Life sciences study design

All studies must disclose on these points even when the disclosure is negative.

Sample size	No statistical methods were used to pre-determine sample size. The sample size was determined according to previous studies (PMID: 27249678, PMID: 29531031)
Data exclusions	Animals in which histological examination showed that viral targeting or the position of cannulas were in the incorrect location were excluded from analysis.
Replication	The experiment for each study was successfully repeated for at least two times.
Randomization	All samples used in the study were randomly allocated into different experimental groups.
Blinding	The data collection and analysis were preformed by investigators blinded to the group allocation.

Reporting for specific materials, systems and methods

We require information from authors about some types of materials, experimental systems and methods used in many studies. Here, indicate whether each material, system or method listed is relevant to your study. If you are not sure if a list item applies to your research, read the appropriate section before selecting a response.

Materials & experimental systems

n/a	Involved in the study	
[ ]	[x]	Antibodies
[x]	[ ]	Eukaryotic cell lines
[x]	[ ]	Palaeontology and archaeology
[ ]	[x]	Animals and other organisms
[ ]	[x]	Human research participants
[x]	[ ]	Clinical data
[x]	[ ]	Dual use research of concern

Methods

n/a	Involved in the study	
[x]	[ ]	ChIP-seq
[x]	[ ]	Flow cytometry
[x]	[ ]	MRI-based neuroimaging

Antibodies

Antibodies used	Immunostaining:<br>Choline Acetyltransferase (ChAT, 1:100; AB144P; Millipore); Tyrosine Hydroxylase (TH; 1:1000; T8700; Sigma); Glutamate (1:1000; G6642; Sigma); GFP (GFP, 1:400; ab13970; Abcam); Living Colors® DsRed Polyclonal Antibody (mCherry, 1:200; 632496; Takara);Donkey anti-Goat Alexa Fluor 488 (1:400; A32814; Invitrogen); Donkey anti-Rabbit Alexa Fluor 488 (1:400; A-21206; Invitrogen); Donkey anti-Rabbit Alexa Fluor 594 (1:400; R37119; Invitrogen); Donkey anti-Chicken Alexa Fluor 488 (1:400; A-11039; Invitrogen); Fluorescent nuclear dye DAPI (F6057; Sigma).<br><br>Western blot:<br>Choline Acetyltransferase (ChAT, 1:1000; AB144P; Millipore) was used as primary antibody. Rabbit Anti-Goat IgG(H+L) HRP-labeled secondary antibody (RAG007, Multi Sciences, China) and Goat Anti-Mouse IgG(H+L) HRP-labeled secondary antibody (GAM007, Multi Sciences, China) were used at a dilution of 1:3000. The antibodies against GAPDH (KC-5G4; KangChen; China) was used for loading controls at a dilution of 1:3000.
Validation	All antibodies used in our study are either validated by the antibody companies.<br>Immunostaining:<br>ChAT: http://www.merckmillipore.com/CN/zh/product/Anti-Choline-Acetyltransferase-Antibody,MM_NF-AB144P?ReferrerURL=https %3A%2F%2Fcn.bing.com%2F&bd=1<br>TH: https://www.sigmaaldrich.com/catalog/product/sigma/t8700?lang=zh&region=CN<br>Glutamate: https://www.sigmaaldrich.com/catalog/product/sigma/g6642?lang=zh&region=CN<br>GFP:https://www.abcam.com/GFP-antibody-ab13970.html<br>mCherry: https://www.labome.com/product/Takara-Bio-Clontech/632496.html<br>Donkey anti-Goat Alexa Fluor 488: https://www.thermofisher.com/antibody/product/Donkey-anti-Goat-IgG-H-L-Highly-Cross-

Adsorbed-Secondary-Antibody-Polyclonal/A32814
Donkey anti-Rabbit Alexa Fluor 488: https://www.thermofisher.com/antibody/product/Donkey-anti-Rabbit-IgG-H-L-Highly-Cross-Adsorbed-Secondary-Antibody-Polyclonal/A-21206
Donkey anti-Rabbit Alexa Fluor 594: https://www.thermofisher.com/cn/zh/antibody/product/Donkey-anti-Rabbit-IgG-H-L-Secondary-Antibody-Polyclonal/R37119
Donkey anti-Chicken Alexa Fluor 488:https://www.thermofisher.com/cn/zh/antibody/product/Goat-anti-Chicken-IgY-H-L-Secondary-Antibody-Polyclonal/A-11039
DAPI: https://www.sigmaaldrich.com/catalog/product/sigma/f6057?lang=zh&region=CN

Western blot:
ChAT: http://www.merckmillipore.com/CN/zh/product/Anti-Choline-Acetyltransferase-Antibody,MM_NF-AB144P?ReferrerURL=https%3A%2F%2Fcn.bing.com%2F&bd=1
Anti-goat IgG, HRP-linked Antibody: http://www.liankebio.com/product-60410.html
Anti-mouse IgG, HRP-linked Antibody: http://www.liankebio.com/product-60308.html
GAPDH: http://www.aksomics.com/products/westernblot-internal-reference-1.html

Animals and other organisms

Policy information about studies involving animals; ARRIVE guidelines recommended for reporting animal research

Laboratory animals	Hrh1fl/fl mice were commercially generated by standard homologous recombination at the Nanjing Biomedical Research Institute of Nanjing University, Nanjing, China. ChAT-Cre mice (stock number: 006410), DAT-Cre mice (stock number: 006660), CaMKIIα-Cre mice (stock number: 005359) and Ai14 mice (stock number: 007914) were genotyped according to the protocols provided by Jackson Laboratories. All mice were bred onto a C57BL/6J genetic background. Male mice 2-4 months of age were used in all experiments. All mice were maintained under a 12 h light-dark cycle (light on from 8:00 a.m. to 8:00 p.m.) with ad libitum access to food and water. All behavior experiments were performed each day between 10:00 a.m. and 7:00 p.m.
Wild animals	This study did not involve wild animals.
Field-collected samples	This study did not involve samples collected from the field.
Ethics oversight	The use and care of the mice were in accordance with the guidelines of the Animal Advisory Committee of Zhejiang University and the US National Institutes of Health Guidelines for the Care and Use of Laboratory Animals. All procedures were approved by the Animal Advisory Committee of Zhejiang University.

Note that full information on the approval of the study protocol must also be provided in the manuscript.

Human research participants

Policy information about studies involving human research participants

Population characteristics	The schizophrenia patients were diagnosed according to the Diagnostic and Statistical Manual of Mental Disorders IV by qualified psychiatrists using the extensive medical records of the NBB, which also contained well-documented diagnoses and onset of schizophrenia from psychiatric clinics. The schizophrenia patients and controls were matched for sex, age, postmortem delay (PMD), clock time and month of death, cerebrospinal fluid (CSF) pH, brain weight and Braak stage of Alzheimer pathology. Detailed clinico-pathological information and p-values of the matched parameter are given in Supplementary Data 1.
Recruitment	The schizophrenia patients recruited were diagnosed according to the Diagnostic and Statistical Manual of Mental Disorders IV by qualified psychiatrists using the extensive medical records of the NBB, which also contained well-documented diagnoses and onset of schizophrenia from psychiatric clinics. Exclusion criteria were any other neurological or psychiatric diseases. Controls had to be non-demented and had no known history of a psychiatric disorder. The schizophrenia patients and controls were matched for sex, age, postmortem delay (PMD), clock time and month of death, cerebrospinal fluid (CSF) pH, brain weight and Braak stage of Alzheimer pathology. These procedures were performed by a staff member of the NBB who was not involved in the experiments in order to prevent any bias.
Ethics oversight	All study protocols were complied with the guidelines for the conduct of research involving human subjects as established by the Ethics Committee of Zhejiang University School of Medicine. All procedures were approved by the Ethics Committee of Zhejiang University School of Medicine.

Note that full information on the approval of the study protocol must also be provided in the manuscript.
